# Electron Redistribution by Fluorine-Induced Dual Defects in Cu_3_P Accelerated Charge Transfer Toward High-Performance Electrochemical Chloride Ion Removal

**DOI:** 10.1007/s40820-026-02267-9

**Published:** 2026-06-23

**Authors:** Ziqing Zhou, Yifan Ren, Fei Yu, Jie Ma

**Affiliations:** 1 State Key Laboratory of Pollution Control and Resource Reuse, Research Center for Environmental Functional Materials, College of Environmental Science and Engineering, Tongji University, 1239 Siping Road, Shanghai, 200092 People’s Republic of China; 2 Xinjiang Key Laboratory of Synthesis and Application of Carbon Nanomaterials, Water Resources and Water Environment Engineering Technology Center, School of Civil Engineering, Kashi University, Kashi, 844000 People’s Republic of China; 3 College of Oceanography and Ecological Science, Shanghai Ocean University, No 999, Huchenghuan Road, Shanghai, 201306 People’s Republic of China

**Keywords:** Fluorine doping, Phosphorus vacancy, Copper(I) phosphide, Electron redistribution, Chloride ion removal

## Abstract

Electrochemical chloride ion removal is essential for clean water and environmental protection, yet its practical application is hindered by the sluggish kinetics, especially using high-mass-loading electrodes. Conventional extrinsic modifications, such as conductive additives or structural design, exhibit constrained effectiveness. Here, we report an intrinsic enhancement strategy through heteroatom doping-induced dual defects engineering, demonstrated by the successful synthesis of fluorine-doped copper(I) phosphide with phosphorus vacancies (F-Cu_3_P_V_) via molten salt treatment. Based on density functional theory calculations and experimental results, F doping caused lattice distortion, generating P vacancies to form dual defects. These defects effectively modulated intrinsic electron redistribution, resulting in improved electrical conductivity, enhanced adsorption capability, and reduced chloride ion diffusion energy barriers. Therefore, electron transfer and ion diffusion kinetics were significantly accelerated, leading to superior electrochemical performance. Resultantly, the F-Cu_3_P_V_ electrode performed exceptional electrochemical chloride ion removal performance with superior areal deionization capacity (3.16 ± 0.02 mg cm^−2^) and a remarkably rapid areal deionization rate (0.106 ± 0.001 mg cm^−2^ min^−1^), as well as outstanding cycling stability (95.65% retention after 70 cycles). This work elucidates electron redistribution via heteroatom doping-induced dual defects as a viable pathway to overcome the intrinsic kinetic bottleneck for high-performance electrochemical chloride ion removal.

**Supplementary Information:**

The online version contains supplementary material available at 10.1007/s40820-026-02267-9.

## Highlights

- Heteroatom doping-induced vacancy was achieved by molten salt treatment.
- The dual defects led to marked electron redistribution, accelerating electron transfer and ion diffusion kinetics.
- The electrodes with dual defects exhibited superior areal deionization capacity and rate.

**Supplementary Information:**

The online version contains supplementary material available at 10.1007/s40820-026-02267-9.

## Graphical abstract

**Figure:**
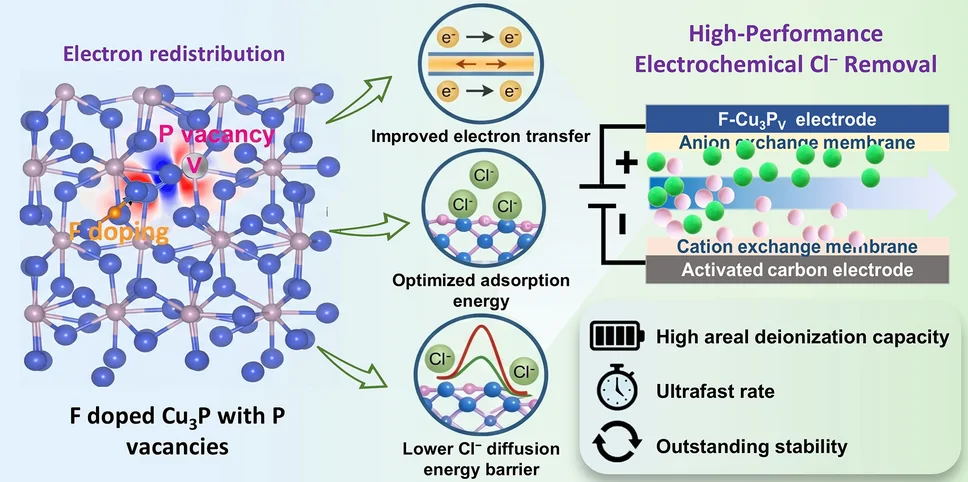

## Introduction

Amid the intensifying global energy crisis and escalating environmental challenges, there is an increasing demand for Faradaic electrode materials with characteristics of exceptional theoretical capacity and large energy density for applications in renewable energy and environmental technologies [1, 2]. Nevertheless, the inherently sluggish reaction kinetics of Faradaic materials limits their performance and seriously hinders their application, particularly as high-mass-loading electrodes (> 10 mg cm^−2^) required for practical commercialization [3]. For high-mass-loading electrodes, the rising mass loading inevitably leads to the increase in thickness of electrode, which would prolong ion and electron transfer path and obstruct the ion channel, resulting in sluggish reaction kinetics [4–6].

To address the aforementioned bottleneck issue, current research efforts predominantly focus on two extrinsic approaches to improve electron transfer and ion diffusion kinetics. The first involved incorporating highly conductive components to accelerate electron transfer and thereby improve electrochemical performance [6–9]. The second focused on structural engineering to optimize ion diffusion dynamics, enabling superior reaction kinetics [9–11]. In our previous study, the low-tortuous copper(I) phosphide (Cu_3_P) nanorod arrays were grown on copper foam through nanostructure engineering to endow ion diffusion and facile one-dimensional electron transfer, thus facilitating accelerated chloride ion (Cl^−^) removal rates [12]. However, the kinetic improvements achievable through these extrinsic approaches remain constrained, primarily because they cannot fully circumvent the intrinsic limitations of the electrode materials themselves, such as inherent poor conductivity and high ion diffusion barriers.

Defect engineering serves as a powerful intrinsic strategy to modulate the intrinsic properties of electrode materials to unlock exceptional electrochemical performance [13, 14]. To date, significant research efforts have focused on utilizing defect engineering to endow electron redistribution for boosting electrochemical kinetics [15, 16]. For instance, phosphorus (P) vacancy can rationally adjust the intrinsic electron distribution to facilitate charge transfer and ion storage, thereby improving the electrochemical performance [17–19]. However, in Cu_3_P, the covalent bonding resulting from the comparable electronegativities of copper (Cu) and P renders bond cleavage challenging [20]. Consequently, developing effective methods to controllably introduce P vacancies into Cu_3_P is crucial for achieving beneficial electron redistribution and thereby enhanced reaction kinetics. Heteroatom doping has been well-established to induce lattice strain and mismatch, substantially promoting the formation of in-plane topological defects, including vacancies, dislocations, and boundaries [21, 22]. These defects locally perturb Gaussian curvature, altering bond configurations and triggering pronounced electron redistribution [23]. A precedent research example by Kou et al. [24] elegantly exploited higher electronegativity difference of nitrogen (3.04) than that of sulfur (2.58) to introduce anion vacancies via nitrogen doping. Inspired by this, fluorine (F) (3.98) possessing the highest electronegativity, exhibits a promising candidate for introducing P vacancy in Cu_3_P based on the heteroatom doping-induced adaptive dual defects engineering, triggering intrinsic electron redistribution to improve Cl^−^ removal kinetics. Despite the considerable theoretical promise, experimental validation remains an unexplored research frontier.

In this work, we proposed a defect engineering strategy involving heteroatom doping to trigger the self-adaptive formation of vacancies, thereby manipulating the intrinsic electron redistribution of Cu_3_P nanorod arrays to optimize their reaction kinetics as Cl^−^ removal electrodes. F-doped Cu_3_P nanorod arrays with P vacancy (F-Cu_3_P_V_) were successfully constructed via low-temperature phosphating combined with controlled molten salt treatment. Calculation and experimental results demonstrated that the lattice distortion induced by F atom incorporation effectively promoted P vacancy formation. The resultant dual defects synergistically regulated electron distribution and optimized the Cl^−^ capture process, leading to significantly enhanced Cl^−^ removal performance. Density functional theory (DFT) calculations were used to elucidate the formation mechanism of the dual defects, their influence on electron distribution, and their critical role in optimizing the Cl^−^ removal process. In conclusion, this study clarifies the enhancement mechanism of F doping and P vacancy dual defects in Cu_3_P for improved Cl^−^ removal performance, offering a viable strategy to optimize the kinetics of Cl^−^ removal electrodes.

## Experimental Section

### Materials

The copper foam was provided by the SCI Materials Hub. Other chemicals and reagents, including hydrochloric acid (HCl, 35 wt%), sodium hydroxide (NaOH), sodium hypophosphite (NaH_2_PO_2_), sodium chloride (NaCl), etc., were provided by Sinopharm Chemical Reagent Co., Ltd. (China).

### Material Preparation

#### ***Synthesis of Monolithic Copper(I) Phosphide Nanorod Arrays (mCu***_***3***_***P NA)***

A piece of copper foam (1.5 cm × 1 cm × 0.8 mm) was first cleaned using 1 M HCl, ethanol, and deionized water via ultrasonication to remove surface impurities. The foam was then dried in the vacuum oven at 60 °C for 12 h. Subsequently, the copper foam was electrochemically anodized at current of 20 mA for 10 min with 3 M NaOH as electrolyte solution, forming of well-ordered Cu(OH)_2_ nanowire arrays. The mCu_3_P NA was synthesized through a low-temperature phosphating process. In this process, the NaH_2_PO_4_ was placed in the center of the tube furnace with Cu(OH)_2_ nanowire arrays placed at the downstream side. At an atmosphere of Ar, the center of the furnace was elevated to 300 °C with a heating rate of 2 °C min^−1^, followed by holding for 120 min. After naturally cooling to room temperature under Ar, the mCu_3_P NA was obtained.

#### ***Synthesis of F-Cu***_***3***_***P***_***V***_

The molten salt treatment was subsequently employed to prepare a series of F-Cu_3_P_V_ materials. In detail, an excess of ammonium fluoride (NH_4_F) was heated to 150 °C until completely melted. The as-synthesized mCu_3_P NA was immersed in the molten salt for specific durations, and the products were removed, washed thoroughly, and finally dried in the vacuum oven. The products treated for 10, 20, and 50 min were denoted as F-Cu_3_P_V_-1, F-Cu_3_P_V_-2, and F-Cu_3_P_V_-3, respectively.

## Results and Discussion

### DFT Preanalysis

**Fig. 1:**
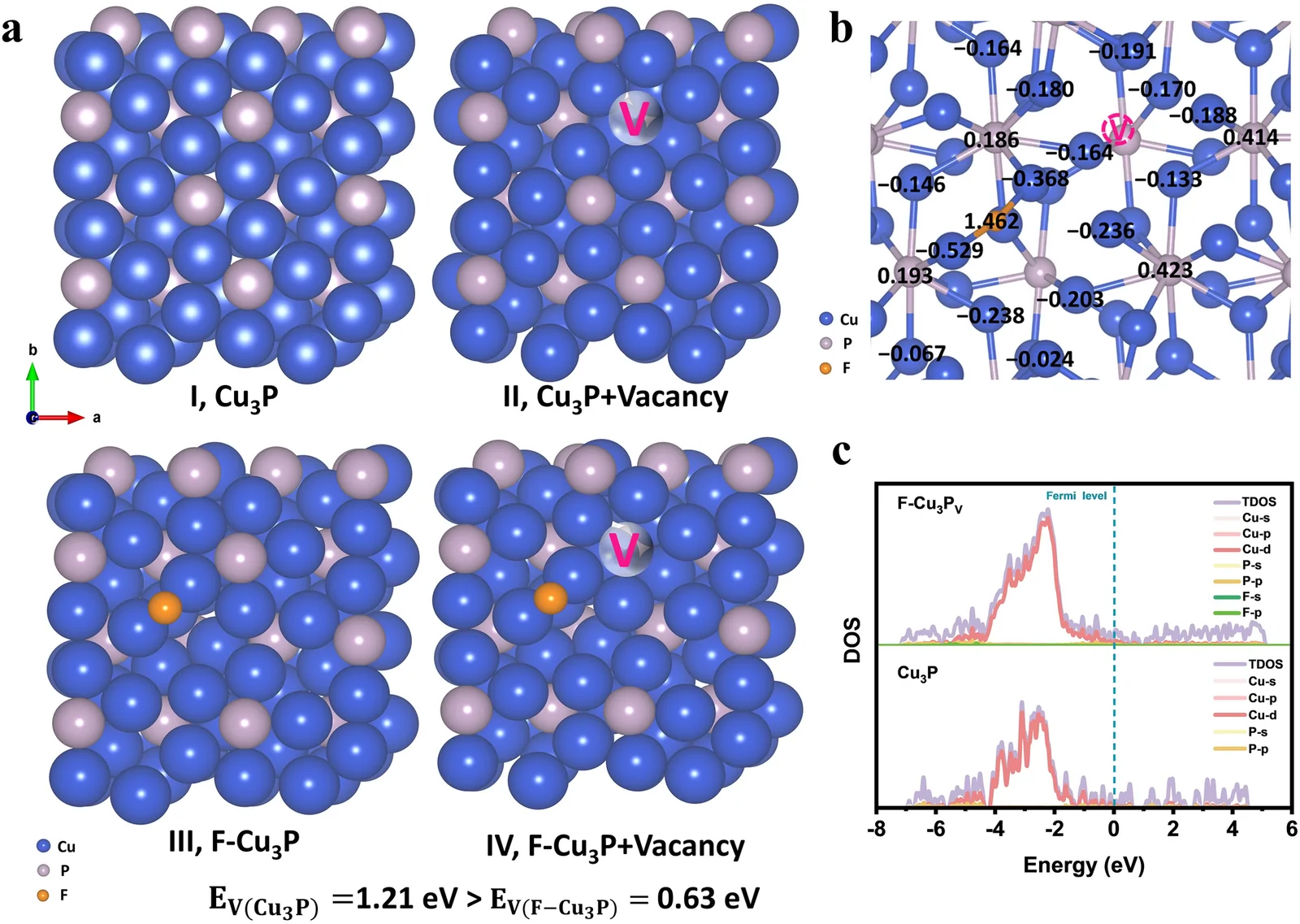
**a** Structural models of Cu_3_P, Cu_3_P_V_, F-Cu_3_P, and F-Cu_3_P_V_ with corresponding E_V_. **b** The Bader charge numbers of atoms in F-Cu_3_P_V_ (Positive and negative values represent the accumulation and depletion of electrons, respectively.). **c** Calculated DOS of Cu_3_P and F-Cu_3_P_V_

To elucidate the influence of F doping on P vacancy formation, DFT calculations were employed to determine the formation energy of P vacancy (*E*_V_). As presented in the computational models (Fig. 1a), the P vacancies were constructed on the pristine Cu_3_P and F-doped Cu_3_P (F-Cu_3_P) models, yielding the corresponding defective configurations (Cu_3_P_V_ and F-Cu_3_P_V_ models). The calculated *E*_V_ for F-Cu_3_P (0.63 eV) was significantly lower than that for Cu_3_P (1.21 eV), demonstrating that the F doping led to the surface reconstruction, which facilitated the formation of P vacancy [23, 25]. Additionally, the effect of the dual defects on electron distribution was subsequently investigated through the Bader charge analysis and density of state (DOS) calculations. The Bader charge for Cu_3_P and F-Cu_3_P_V_ revealed that the F doping and P vacancy led to a substantial redistribution of electrons (Figs. 1b and S1) [18, 26]. Specifically, after introducing the F atom and P vacancy, the charge density around the F atom increased due to its strong electron-withdrawing nature, which further affected electron distribution of Cu and P atoms [27, 28]. Furthermore, compared to Cu_3_P model, the F-Cu_3_P_V_ model exhibited an increased density of electronic states near the Fermi level (Fig. 1c), confirming a charge-localized redistribution with improved electrical conductivity [29]. This can effectively accelerate intrinsic charge-transfer kinetics of F-Cu_3_P_V_ in deionization process [30]. Further analysis of models containing only F doping or only a P vacancy indicates that single defect can also induce electronic redistribution and improve conductivity, but the dual defects exhibit a more pronounced optimized effect (Figs. S2 and S3). In conclusion, the introduction of F atom would favor the formation of P vacancy, thus achieving the electron redistribution of F-Cu_3_P_V_ electrodes, showing the superior potential to improve the intrinsic electron transfer and ion diffusion kinetics during deionization process.

### Synthesis and Characterization

**Fig. 2:**
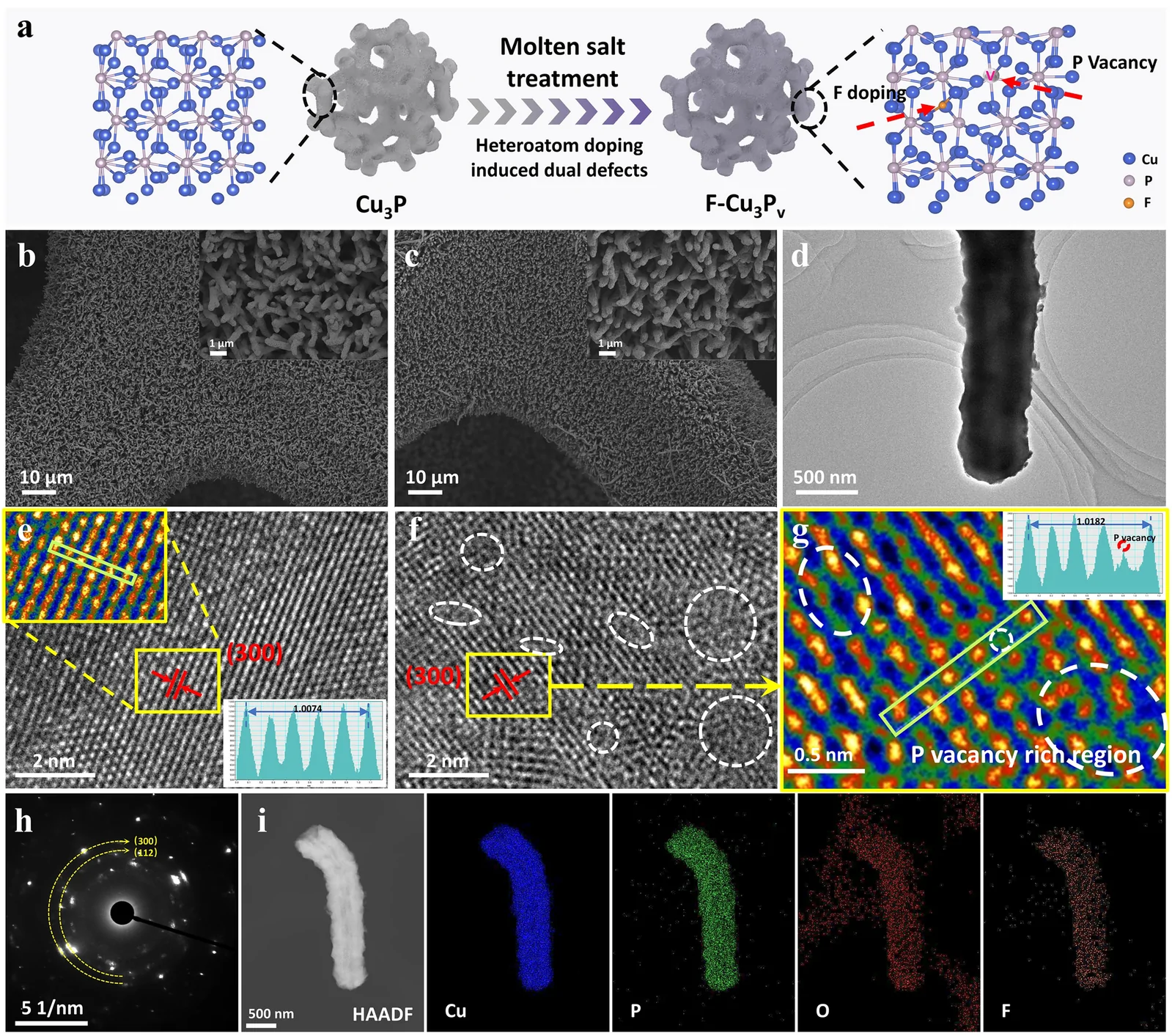
**a** Schematic illustration of the synthesis process of F-Cu_3_P_V_ samples. SEM images at different magnifications of **b** mCu_3_P NA and **c** F-Cu_3_P_V_-2. **d** TEM image of F-Cu_3_P_V_-2. HRTEM of **e** mCu_3_P NA (inset images were false-color HRTEM image of the areas marked with a yellow rectangle in TEM image and corresponding line profile extracted from the areas marked with a green rectangle in TEM image) and **f** F-Cu_3_P_V_-2. **g** False-color HRTEM image of F-Cu_3_P_V_-2 (inset image was corresponding line profile extracted from the areas marked with a green rectangle in TEM image). **h** SEAD of F-Cu_3_P_V_-2. **i** High-angle annular dark field (HAADF) scanning TEM image of F-Cu_3_P_V_-2 and corresponding EDS element mappings

The F-Cu_3_P_v_ was synthesized through the strategy of low-temperature phosphating and molten salt treatment. The synthesis process is displayed in Figs. S4 and 2a. Firstly, the monolithic low-tortuous Cu_3_P nanorod arrays (mCu_3_P NA) were fabricated by the low-temperature phosphating with Cu(OH)_2_ nanowire arrays on copper foam (Fig. S5) as precursors [12]. The uniformly distributed nanorod array structure was demonstrated in the scanning electron microscopy (SEM) image (Fig. 2b). Subsequently, the F-Cu_3_P_V_ samples were synthesized by subjecting the above-obtained mCu_3_P NA to the molten salt treatment, during which the morphology of nanorod arrays was well maintained (Fig. 2c). In detail, the mCu_3_P NA was immersed in molten NH_4_F for varying durations [30, 31]. The F-Cu_3_P_V_ samples were designated F-Cu_3_P_V_-1, F-Cu_3_P_V_-2, and F-Cu_3_P_V_-3 with treatment times of 10, 20, and 50 min, respectively. The transmission electron microscopy (TEM) image was shown in Figs. 2d and S6a to confirm the unchanged rod structure after treatment, indicating that F doping and P vacancy would not affect the morphology. The high-resolution TEM (HRTEM) image demonstrated that nanorod of mCu_3_P and F-Cu_3_P_V_-2 was mainly Cu_3_P, with an interplanar spacing of about 0.20 nm assigned to (300) plane of hexagonal Cu_3_P phase (JCPDS 71–2261) (Fig. 2e, f) [12]. Nevertheless, in comparison with mCu_3_P NA, the lattice stripe of F-Cu_3_P_V_-2 exhibited obvious discontinuities (marked with white circles), which can be attributed to the heteroatom F doping and the formation of P vacancies [26, 30, 32]. Furthermore, the evident absence of atoms could be observed in the circle areas in the false-color HRTEM image of F-Cu_3_P_V_-2, confirming the existence of the vacancies [33–36]. The formation of P vacancy was additionally supported by the corresponding line profiles extracted from the selected area in HRTEM of F-Cu_3_P_V_-2 [17, 18, 37]. Notably, after molten salt treatment, the lattice spacing of (300) plane increased from 0.201 nm in mCu_3_P NA to 0.204 nm in F-Cu_3_P_V_-2. This expansion can be attributed to the introduction of doping and vacancy, which caused the surrounding atoms to converge or stretch due to the differences in radius and electronegativity, leading to lattice distortion [18, 37]. As displayed in the selected area electron diffraction (SAED) patterns of mCu_3_P and F-Cu_3_P_V_-2 (Figs. S6b and 2h), apparent diffraction rings were assigned to (300) plane and (112) plane of Cu_3_P, which revealed that the molten salt treatment would not change the crystal phase. The energy-dispersive X-ray spectroscopy (EDS) elemental mappings illustrated the uniform distribution through the whole rod in mCu_3_P NA and F-Cu_3_P_V_-2 (Figs. S6c and 2i). And the appearance of F element in F-Cu_3_P_V_-2 supported the successful doping of F.

**Fig. 3:**
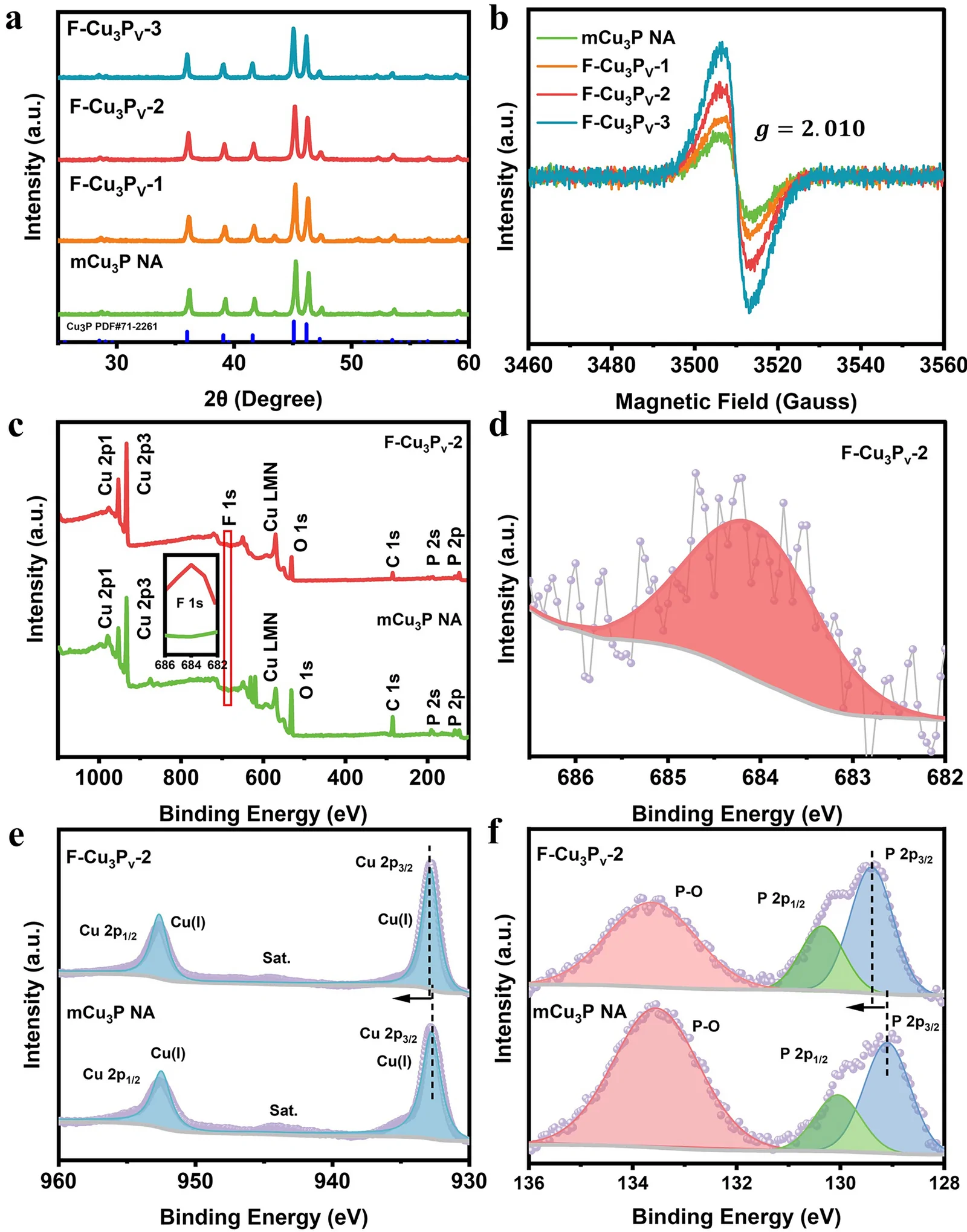
**a** XRD patterns and **b** EPR spectra of mCu_3_P NA and three F-Cu_3_P_V_ samples. **c** XPS survey of mCu_3_P NA and F-Cu_3_P_V_-2. **d** High-resolution XPS of F 1*s* of F-Cu_3_P_V_-2. High-resolution XPS of **e** Cu 2*p* and **f** P 2*p* of mCu_3_P NA and F-Cu_3_P_V_-2

The X-ray diffraction (XRD) patterns in Fig. 3a illustrated the characteristic peaks of mCu_3_P NA and F-Cu_3_P_V_ well matched with standard Cu_3_P (JCPDS 71-2261), suggesting that molten salt treatment did not change the crystal phase. Notably, compared with that of mCu_3_P NA, the characteristic peaks of three F-Cu_3_P_V_ samples exhibited low-angle shifts, and as the treatment time increased, the shift became more obvious. This demonstrated that the dual defects of F doping and P vacancies would enlarge the lattice space, consistent with the result of HRTEM results. To verify the presence of P vacancies in F-Cu_3_P_V_, the electron paramagnetic resonances (EPR) were carried out. Compared to mCu_3_P NA with weak symmetric signals, F-Cu_3_P_V_ possessed strong symmetric signals with a *g*-value of 2.010, confirming the existence of unpaired electrons attributed to P vacancies (Figs. 3b and S7) [17, 27]. This further supported that the heteroatom F doping induced the rich P vacancies during the molten salt treatment. Furthermore, as the treatment time increased, the intensity of signals was promoted, suggesting a positive correlation between the concentration of P vacancy and duration of molten salt treatment. To investigate the surface composition and further verify F doping and P vacancies, X-ray photoelectron spectroscopy (XPS) was conducted. The XPS survey (Fig. 3c) revealed the existence of Cu and P elements on the surface of mCu_3_P NA and F-Cu_3_P_V_-2. Besides, the introduction of F was further verified by the XPS survey result, consistent with the EDS mapping results. As shown in F 1*s* high-resolution XPS spectrum (Fig. 3d), a characteristic peak at 684.08 eV assigned to the F-Cu bonds was detected on the F-Cu_3_P_V_-2, suggesting successful doping of F [31]. The Cu 2*p* XPS of mCu_3_P NA and F-Cu_3_P_V_-2 revealed a slight positive shift of approximately 0.1 eV in the binding energy of peaks corresponding to Cu^+^, suggesting that the dual defects resulted in the electronic redistribution (Fig. 3e). Specifically, according to the transfer characteristics of electrons, the heteroatom F with stronger electronegativities caused electrons to transfer from Cu to F, resulting in the positive shift in binding energy [38]. In addition, the binding energy of P 2*p* for F-Cu_3_P_V_-2 was higher than that for mCu_3_P NA (0.3 eV), indicating the occurrence of electron transfer between Cu and P (Fig. 3f). As discussed above, the molten salt treatment achieved the doping of F with strong electronegativity, promoting the electron transfer from Cu to F. This led to the weakening of Cu-P bonds, thus inducing the generation of P vacancies to form the dual defects on F-Cu_3_P_V_. Furthermore, the adaptive dual defects of F doping and P vacancies endowed the electron redistribution in F-Cu_3_P_V_, which would effectively favor high-performance Cl^−^ removal.

**Fig. 4:**
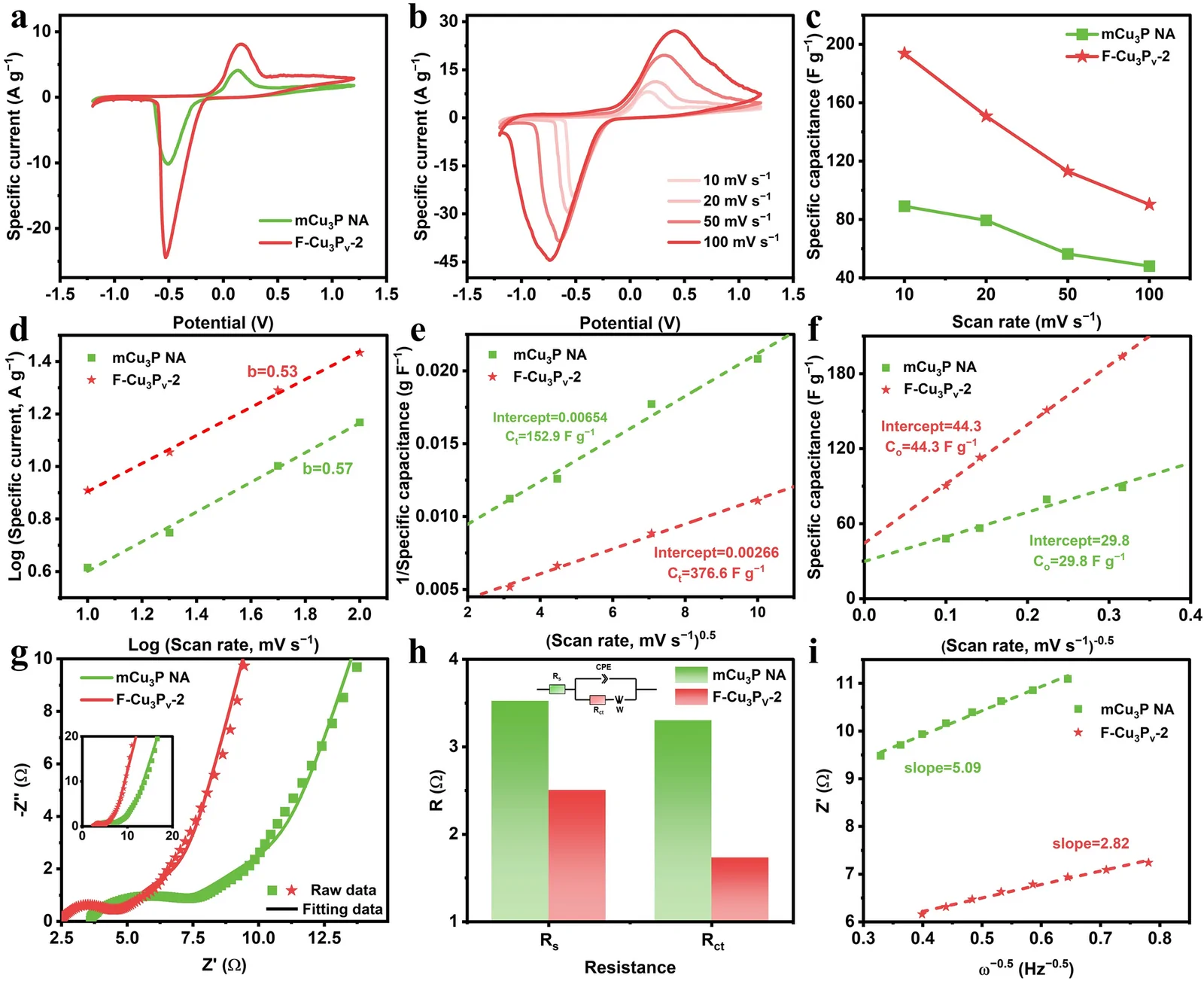
**a** CV curves of mCu_3_P NA and F-Cu_3_P_V_-2 at scan rate of 10 mV s^−1^. **b** The CV curves of F-Cu_3_P_V_-2 at different scan rates. **c** Specific capacitances of mCu_3_P NA and F-Cu_3_P_V_-2 at different scan rates. **d** Power-law relationship between specific current and scan rate. Plot of **e** 1/C versus ${v}^{0.5}$ and **f** C versus ${v}^{-0.5}$. **g** Nyquist plots obtained from EIS and **h** corresponding *R*_s_ and *R*_ct_ (inset image was the equivalent electric circuit diagram). **i** Linear fitting result between *Z*’ and *ω*^−0.5^ in the low-frequency region

The effect of electron redistribution originating from adaptive dual defects of F doping and P vacancy on the electrochemical performance was explored by utilizing a series of electrochemical investigations in a three-electrode setup with 1 M NaCl as electrolyte solution. The cyclic voltammetry (CV) curves of all samples exhibited a pair of obvious redox peaks, suggesting that the capture and release of Cl^−^ by Cu_3_P were realized by the redox reactions (${\mathrm{C}\mathrm{u}}_{3}\mathrm{P} +3{\mathrm{C}\mathrm{l}}^{-}\leftrightarrow 3\mathrm{C}\mathrm{u}\mathrm{C}\mathrm{l}+\mathrm{P}+3{\mathrm{e}}^{-})$ (Figs. 4a and S8). This was consistent with the previous research [12]. Moreover, all F-Cu_3_P_V_ samples displayed higher specific current in CV profile than mCu_3_P NA, and the F-Cu_3_P_V_-2 offered the largest integrated area of the CV curves at different scan rates, indicating the highest specific capacitance (Figs. 4b and S9). In addition, the calculated results of the specific capacitance are presented in Figs. 4c and S10. Obviously, the F-Cu_3_P_V_ samples possessing the dual defects exhibited higher specific capacitance than mCu_3_P NA. Specifically, at the scan rate of 10 mV s^−1^, the specific capacitance of F-Cu_3_P_V_-2 was up to 193.7 F g^−1^, which was twice that of mCu_3_P NA (89.05 F g^−1^). These results revealed that the adaptive dual defects of F doping and P vacancy could enhance electrochemical activity to promote deionization performance. The calculated *b* values based on the power-law relationship ($i=a{v}^{b}$, where i denotes the current, v is scan rate) of mCu_3_P NA and F-Cu_3_P_V_-2 were 0.57 and 0.53, respectively, confirming the charge storage process dominated by diffusion behavior (Fig. 4d). Additionally, the Trasatti method was further employed to investigate the impact of dual defects on charge storage process [39]. The total capacitance (${C}_{t}$) was composed of outer surface capacitance (${C}_{\mathrm{o}}$) and inner surface capacitance (${C}_{\mathrm{i}}$), which can be determined using the equation ${C}_{t}\left(v\right)=C+k{v}^{-0.5}$, where k is a constant and v represents the scan rate. Linear fitting of 1/C and C versus ${v}^{0.5}$ and ${v}^{-0.5}$ allowed for the extrapolation of C to v=0 and $v\to \infty$, facilitating the determination of ${C}_{t}$ and ${C}_{\mathrm{o}}$, respectively (Fig. 4e-f). The values obtained for ${C}_{\mathrm{t}}$ were 152.9 F g^−1^ for mCu_3_P NA and 376.6 F g^−1^ for F-Cu_3_P_V_-2, with corresponding ${C}_{\mathrm{o}}$ values of 44.3 and 29.8 F g^−1^. Obviously, the dual defects in F-Cu_3_P_V_-2 effectively promoted the charge storage to improve the electrochemical performance, thus endowing the potential to achieve high deionization performance [40]. Moreover, the greater contribution of ${C}_{\mathrm{i}}$ compared to ${C}_{\mathrm{o}}$ in both samples confirmed that the diffusion-controlled process dominated the charge storage in Cu_3_P. The significant enhancement of ${C}_{\mathrm{i}}$ from 123.1 F g^−1^ for mCu_3_P NA to 332.3 F g^−1^ for F-Cu_3_P_V_-2, revealed that the F doping and P vacancy enriched the active sites for redox reaction and promoted inner charge storage to improve diffusive contribution, thus boosting the overall electrochemical performance [41–43].

Electrochemical impedance spectroscopy (EIS) results were fitted with the equivalent circuit to investigate the impact of adaptive dual defects on the charge storage kinetics, as illustrated in Fig. 4g, h. Obviously, the series resistance (*R*_s_) of F-Cu_3_P_V_-2 (2.50 Ω) was lower than that of mCu_3_P NA (3.52 Ω), thus improving electron transfer. Additionally, F-Cu_3_P_V_-2 possessed a markedly reduced charge-transfer resistance (*R*_ct_) of 1.73 Ω compared to mCu_3_P NA of 3.30 Ω, implying improved charge-transfer kinetics. In addition, the real capacitance and imaginary capacitance were derived from EIS data (Fig. S11). In the low-frequency region, the F-Cu_3_P_V_-2 exhibited greater capacitances and accelerated ion diffusion, as evidenced by the minimum relaxation time constant τ_0_ of F-Cu_3_P_V_-2 (31.63 s) compared with that of mCu_3_P NA (38.31 s) [44]. The enhanced ion diffusion kinetics were further corroborated by the Warburg coefficient (*σ*) obtained from the slope of the plot of *Z*′ versus *ω*^−0.5^. As shown in Fig. 4i, the introduction of the adaptive dual defects resulted in a decrease in σ from 5.09 for mCu_3_P NA to 2.82 for F-Cu_3_P_V_-2, confirming the faster ion diffusion kinetics. These findings underscored that the incorporation of adaptive dual defects in F-Cu_3_P_V_-2 significantly enhanced both electron transfer and ion diffusion kinetics, thereby improving deionization kinetics.

**Fig. 5:**
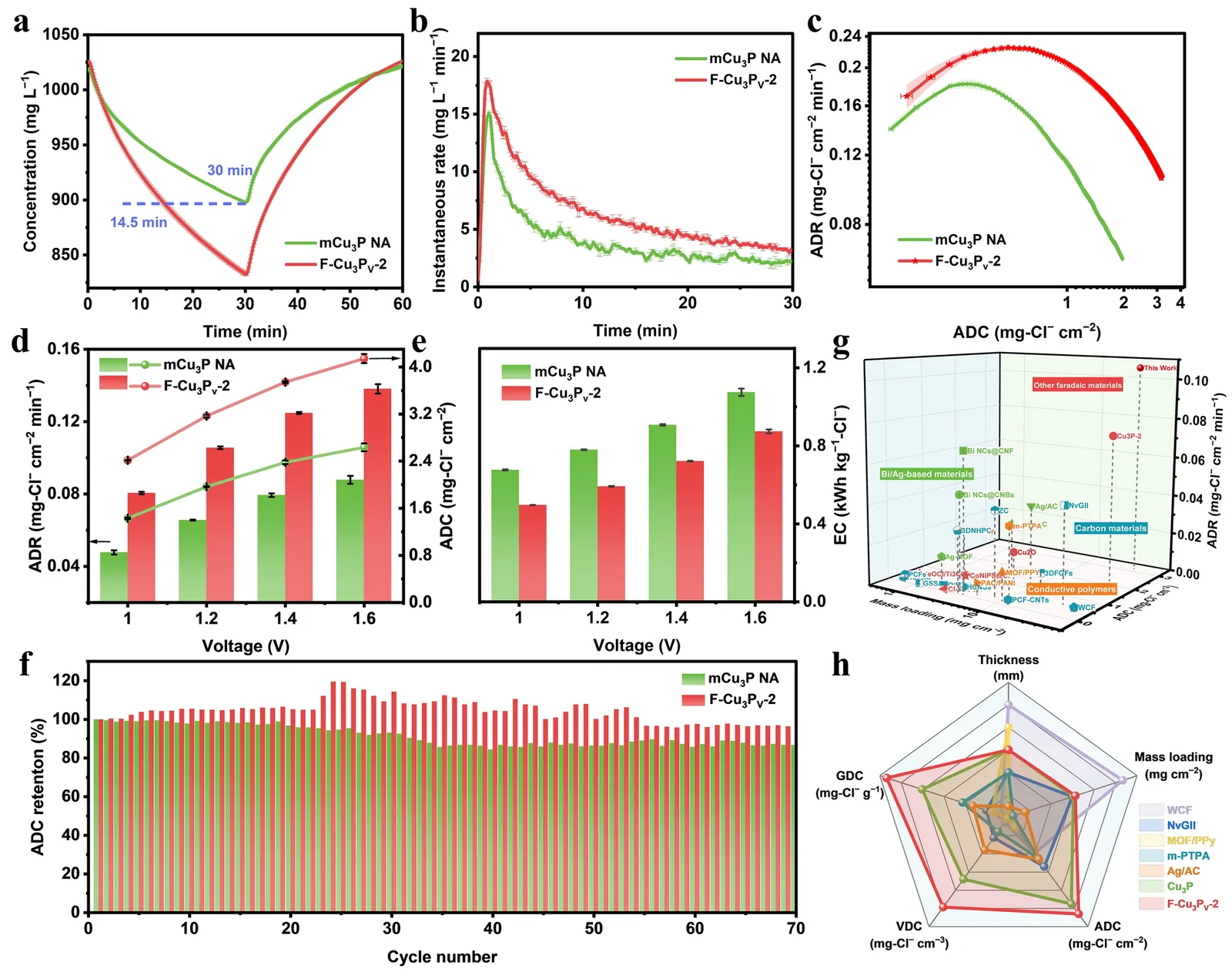
Temporal variation in **a** concentration of NaCl solution, **b** the corresponding instantaneous rate of concentration decreasing, and **c** Ragone plots of ADC and ADR of mCu_3_P NA and F-Cu_3_P_V_-2 operating at 1.2 V. Comparison of **d** ADC and ADR as well as **e** EC with operation voltages ranging from 1.0 to 1.6 V. **f** ADC retention during the long-term cycles. **g** Comparison of mass loading, ADC, and ADR of F-Cu_3_P_V_-2 with the other state-of-the-art Cl^−^ removal electrode. **h** Rader map comparison of high-mass-loading electrodes

To assess the impact of adaptive dual defects on electrochemical Cl^−^ removal performance, the electrochemical deionization (EDI) system was constructed using prepared materials and activated carbon as anode and cathode, respectively (Fig. S12). Consistent with the previous research about mCu_3_P NA, we employed the areal assessment parameters to evaluate the deionization performance of the prepared electrodes [12]. This approach was taken due to the monolithic nature of the electrodes and the superior capability of areal assessment parameters in evaluating practical-use feasibility. As shown in Fig. S13, all F-Cu_3_P_V_ samples performed higher areal deionization capacity (ADC) than mCu_3_P NA, revealing that adaptive dual defects could effectively enhance the deionization performance. Notably, the F-Cu_3_P_V_-2 exhibited the highest ADC and thus was selected to further analyze the impact of the dual defects. As depicted in Fig. 5a, under a constant voltage of 1.2 V, the NaCl concentration decreased from about 1020 to 897.7 mg L^−1^ within 30 min for mCu_3_P NA, while the F-Cu_3_P_V_-2 achieved a comparable reduction in less than half the time (~ 14.5 min), followed by a concentration decrease in concentration to 832.7 mg L^−1^ at 30 min. This convincingly demonstrated the efficacy of the adaptive dual defects in promoting the electrochemical Cl^−^ removal kinetics. The corresponding instantaneous rates of concentration decrease are presented in Fig. 5b. Obviously, the instantaneous rate of F-Cu_3_P_V_-2 was higher than that of mCu_3_P NA in the whole deionization process, with the highest instantaneous rate of NaCl concentration up to 17.85 ± 0.27 mg L^−1^ min^−1^. Additionally, the variations in time-average areal deionization rate (ADR) versus time also proved the faster kinetics of F-Cu_3_P_V_-2 (Fig. S14). Moreover, as shown in the Ragone plots (Fig. 5c), the plots of F-Cu_3_P_V_-2 were positioned in the more rightward and upper area, revealing the more exceptional performance of F-Cu_3_P_V_-2 with superior ADC (3.16 ± 0.02 mg cm^−2^) and a remarkably rapid ADR (0.106 ± 0.001 mg cm^−2^ min^−1^). In constant voltage operation, applied voltage serves as a critical performance determinant, so deionization tests were conducted at various voltages to assess the maintainability of the performance enhancement from dual defects across different voltages (Fig. 5d). With the voltage increasing from 1.0 to 1.6 V, the ADR and ADC of all samples increased stepwise. Furthermore, the F-Cu_3_P_V_-2 electrode performed higher ADR and ADC at all applied voltages and possessed the highest ADR of 0.138 ± 0.003 mg cm^−2^ min^−1^ with a corresponding ADC of 4.14 ± 0.08 mg cm^−2^ at 1.6 V. The improved ADC of mCu_3_P NA and F-Cu_3_P_V_-2 at increased stride length of 0.2 V were ~ 0.40 and 0.58 mg cm^−2^, respectively, illustrating promoted sensitive response to voltage increase for Cl^−^ capture on F-Cu_3_P_V_-2. In addition, the energy consumptions (EC) of F-Cu_3_P_V_-2 were lower than that of mCu_3_P NA, suggesting that the energy-saving of F-Cu_3_P_V_-2 for ion capture (Fig. 5e). Moreover, F-Cu_3_P_V_-2 exhibits highly competitive energy consumption performance compared to the previously reported electrode (Fig. S15). The superior areal performance of F-Cu_3_P_V_-2 compared to mCu_3_P NA could be attributed to the electron redistribution induced by F doping and P vacancy, which significantly improve intrinsic charge transfer and ion adsorption.

The cycling stability acts as one of significant evaluation indices for assessing the performance of EDI electrodes. The defect engineering has been widely utilized to improve the stability of electrode. As shown in Fig. 5f, the long-term adsorption–desorption cycling tests were conducted in 1000 mg L^−1^ NaCl solution at 1.2 V to investigate the impact of the adaptive dual defect of F doping and P vacancy on the cycling performance. Resultantly, the F-Cu_3_P_V_-2 electrode displayed exceptional cycling performance with higher ADC retention of 95.65% over 70 cycles than mCu_3_P NA (86.67%). These results demonstrated that the dual defect can effectively enhance the cycling stability performance. In addition, to further confirm the excellent cycling stability, the characterizations of F-Cu_3_P_V_-2 electrode after long-term cycling were conducted. As shown in Figs. S16 and S17, these results revealed that the nanorod array structure, the crystal phase, the presence of phosphorus vacancies, and the chemical composition were maintained well, evidently supporting the outstanding cycling stability of F-Cu_3_P_V_-2. To further illustrate the superiority of the dual-defect engineering F-Cu_3_P_V_-2 electrode, the Kim-Yoon plots were employed to comprehensively compare the areal deionization performance of recently reported high-performance Cl^−^ removal electrodes with various mass loadings (Fig. 5g). The detailed information is exhibited in Table S1. Notably, the F-Cu_3_P_V_-2 electrode stood out from them owing to its markedly superior ADC and ADR, underscoring its exceptional potential in electrochemical Cl^−^ removal. In addition, high-mass-loading electrodes (> 10 mg cm^−2^) typically possess high areal and volumetric performance, which is conducive to improving areal utilization of the electrode and the overall volumetric deionization efficiency of the EDI device. These attributes align more closely with the demands of practical applications. However, increasing mass loading will inevitably lead to electrode thickening, which extends the charge distance and negatively impacts electrochemical kinetics, ultimately resulting in a marked reduction in gravimetric performance. Therefore, achieving a trade-off between ADC, volumetric deionization capacity (VDC), and gravimetric deionization capacity (GDC) is crucial for advancing the practical application of high-mass-loading electrodes. As illustrated in the radar plot of Fig. 5h, among these high-mass-loading electrodes, the F-Cu_3_P_V_-2 electrode exhibited superior EDI properties, possessing the highest ADC, VDC, and GDC simultaneously (Table S2). The superior performance could be attributed to the improved charge-transfer kinetics by dual defects, which effectively mitigated the sluggish kinetics associated with the increased electrode thickness.

To elucidate the underlying mechanism of enhanced deionization performance observed in F-Cu_3_P_V_ electrodes through electron redistribution induced by dual defects, the DFT calculations were systematically conducted to investigate the Cl^−^ storage behavior of Cu_3_P and F-Cu_3_P_V_ models (Fig. S18). The charge density differences during chloride adsorption in top, front, and corresponding section views are displayed in Figs. S19 and 6a–d. Notably, the F-Cu_3_P_V_ demonstrated a more pronounced charge-transfer behavior compared to the Cu_3_P, suggesting that the electron redistribution induced by the dual defects affected charge-transfer behaviors during adsorption, thus improving adsorption performance [29, 45]. This was further corroborated by the Bader charge analysis. The calculated charge transfer of F-Cu_3_P_V_ (0.83 e) was markedly higher than that of Cu_3_P (0.56 e), indicating an enhanced interaction and ionic bond nature between Cl^−^ and F-Cu_3_P_V_ [46, 47]. Furthermore, the F-Cu_3_P_V_ displayed a more negative adsorption energy (${E}_{\mathrm{a}}$) of − 4.58 eV compared to Cu_3_P (${E}_{\mathrm{a}}= -$ 4.22 eV), suggesting that the electron redistribution induced by F doping and P vacancy endowed with better Cl^−^ adsorption ability. This was thereby favor in providing more active sites and more rapid deionization kinetics [48]. Moreover, the Cl^−^ diffusion process in Cu_3_P and F-Cu_3_P_V_ model were explored (Figs. S20 and 6e, f). It is worth notably that the dual defects of F doping and P vacancy can effectively lower the Cl^−^ diffusion energy barrier (0.38 eV for F-Cu_3_P_V_ and 0.48 eV for Cu_3_P), improving the intrinsic kinetics of ion transfer. In addition, the F-Cu_3_P model exhibits more significant charge transfer, more negative adsorption energy, and lower diffusion energy barrier compared to the Cu_3_P_V_ model, suggesting that F doping shows a greater enhancement on Cl^−^ adsorption and kinetics than P vacancy (Fig. S21). Based on the DFT preanalysis and mechanism discussion, the dual defects of F doping and P vacancy can greatly optimize the electronic distribution of F-Cu_3_P_V_, which accelerated the electron transfer and ion diffusion, thus improving the deionization rate.

**Fig. 6:**
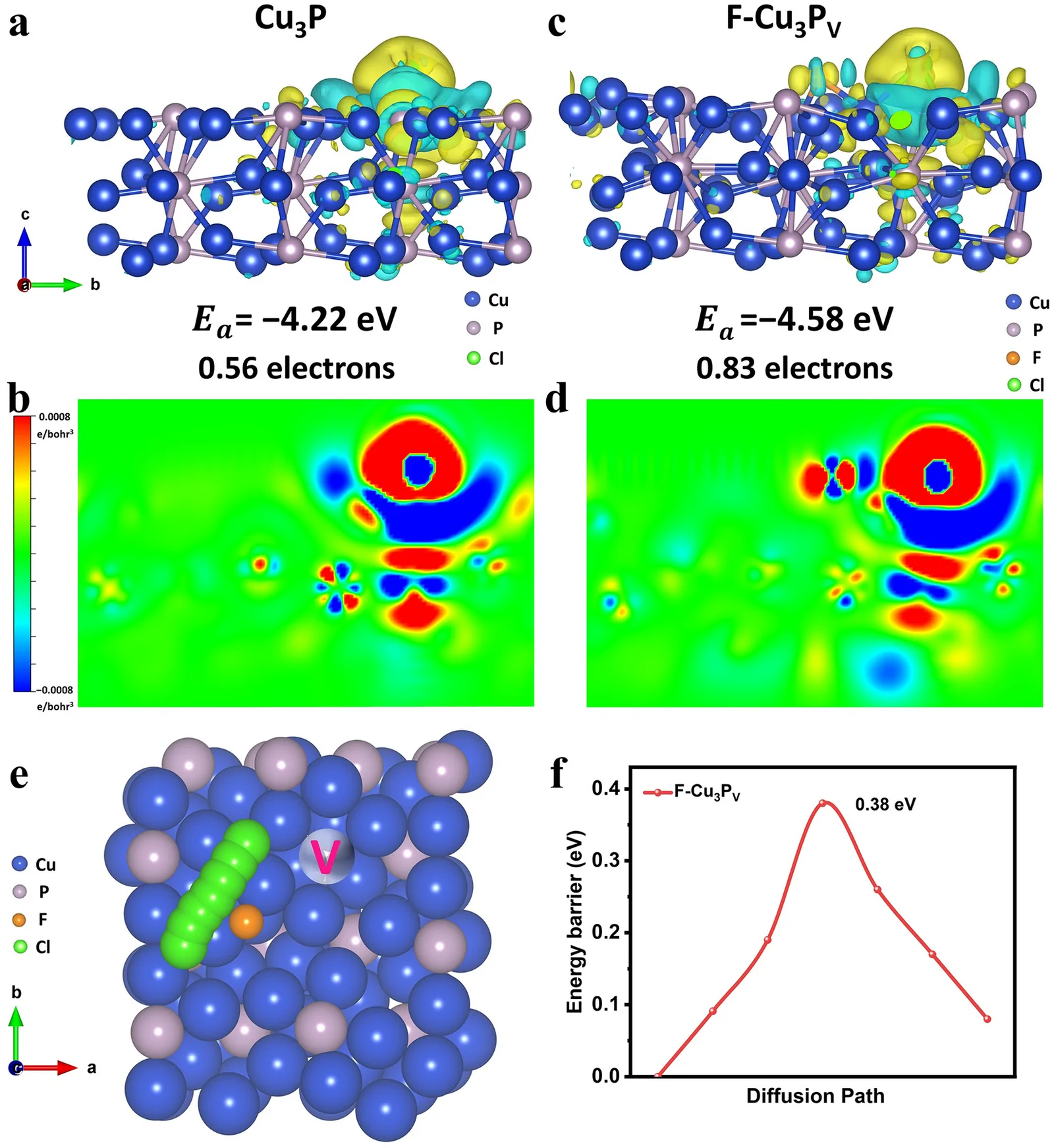
Front and corresponding section views of the charge density difference during the Cl^−^ adsorption for **a-b** Cu_3_P and **c-d** F-Cu_3_P_V_. (Yellow area indicates electron accumulation and green area indicates electron depletion.) **e** Top view of Cl^−^ diffusion path on F-Cu_3_P_V_ and **f** corresponding energy barrier diffusion curves

## Conclusions

In summary, guided by the DFT preanalysis, F-Cu_3_P_V_ samples were constructed by heteroatom doping-induced dual defects through molten salt treatment, thereby improving performance as EDI electrode for Cl^−^ removal. According to experimental and calculation results, lattice distortion resulting from F doping led to the formation of P vacancies, thus modulating electron redistribution. This endowed intrinsic improved conductivity, enhanced adsorption ability, and lower Cl^−^ diffusion energy barrier, which significantly promoted electron transfer and ion diffusion kinetics to improve electrochemical performance. Consequently, F-Cu_3_P_V_-2 electrodes exhibited exceptional Cl^−^ removal performance with superior ADC (3.16 ± 0.02 mg cm^−2^) and a remarkably rapid ADR (0.106 ± 0.001 mg cm^−2^ min^−1^), as well as outstanding cycling stability (95.65% retention after 70 cycles). More importantly, the performance of F-Cu_3_P_V_ electrode was superior to the previously reported Cl^−^ removal electrodes, and achieved great balance of ADC, VDC, and GDC as high-mass-loading electrodes. This work not only introduces a high-mass-loading electrode with superior kinetics, but also provides a new pathway to improve intrinsic kinetics by regulating electron redistribution.

## Supplementary Information

Below is the link to the electronic supplementary material.

Supplementary file1 (DOCX 9782 kb)
