# Supplementary material for: Electron Redistribution by Fluorine-Induced Dual Defects in Cu3P Accelerated Charge Transfer Toward High-Performance Electrochemical Chloride Ion Removal
Source: Nanomicro Lett. 2026 Jun 23;18:412. doi: 10.1007/s40820-026-02267-9 (PMC13287301; doi:10.1007/s40820-026-02267-9)
Supplement: Supplementary file 1 — Supplementary file1 (DOCX 9782 kb) [file 40820_2026_2267_MOESM1_ESM.docx]

Supporting Information for

**Electron Redistribution by Fluorine-Induced Dual Defects in Cu_3_P Accelerated** **Charge Transfer towards High-Performance Electrochemical Chloride Ion Removal**

Ziqing Zhou^1^, Yifan Ren^1^, Fei Yu^3^, Jie Ma^1,2*^

^1^ Research Center for Environmental Functional Materials, State Key Laboratory of Pollution Control and Resource Reuse, College of Environmental Science and Engineering, Tongji University, 1239 Siping Road, Shanghai 200092, P. R. China

^2^ Water Resources and Water Environment Engineering Technology Center, Xinjiang Key Laboratory of Synthesis and Application of Carbon Nanomaterials, School of Civil Engineering, Kashi University, Kashi 844000, P. R. China

^3^ College of Oceanography and Ecological Science, Shanghai Ocean University, No 999, Huchenghuan Road, Shanghai, 201306, P. R. China

^*^Corresponding author. E-mail: jma@tongji.edu.cn (Jie Ma)

**S1 Experimental**

**S1.1 Characterization**

The morphological characteristics of the samples were investigated using scanning electron microscopy (SEM, Hitachi S-4800, Japan) and transmission electron microscopy (TEM, JEOL JEM-F200). The crystal structure was analyzed via X-ray diffraction (XRD, Bruker D2 PHASER). Furthermore, the oxidation states and surface chemical composition of the composites were examined by X-ray photoelectron spectroscopy (XPS, Thermo Scientific ESCALAB 250Xi).

**S1.2 Electrochemical measurements**

The electrochemical tests were performed in 1 M NaCl electrolyte using a three-electrode electrochemical cell, comprising a platinum (Pt) sheet as counter electrode, a silver/silver chloride (Ag/AgCl) electrode as reference electrode and prepared samples as working electrodes. An electrochemical workstation (CHI660D, Chenhua Instruments Co., China) was utilized to conduct cyclic voltammetry (CV) and electrochemical impedance spectroscopy (EIS) measurements. For CV tests, the scan rates were conducted at 10, 20, 50, and 100 mV s^−1^ within a voltage window of −1.2 ~ 1.2 V. The EIS analysis was performed over a frequency range of 0.1 Hz to 100 kHz at the open-circuit potential.

The gravimetric special capacitance obtained from CV curve was calculated according to equation (S1).

$$\begin{aligned} C=\frac{\int idV}{2mv\Delta V}\#\left( S1 \right) \end{aligned}$$

Where C represents the specific capacitance (F g^−1^), *i* refers to the current (A), *v* is the scan rate (V s^−1^), *ΔV* is the potential window (V), *m* corresponds the electrode material mass (g).

The power law relationship between the current and scan rates of CV plots conforms the follow equation (S2).

$$\begin{aligned} i=av^{b}\#\left( S2 \right) \end{aligned}$$

where $i$ denotes the current (A), $v$ is scan rate (mV s^−1^), $a$ and $b$ are constant value.

The Trasatti analysis method was utilized to assess the capacitance contributions, and the calculations were based on the following equations (S3) and (S4).

$$\begin{aligned} C_{t}\left( v \right)=C_{O}+kv^{-0.5}\#\left( S3 \right) \end{aligned}$$

$$\begin{aligned} C_{t}=C_{o}+C_{i}\#\left( S4 \right) \end{aligned}$$

Where $C_{t}\left( v \right)$ is the voltametric capacitance, $C_{t}$ is the total capacitance, $C_{o}$ is the outer capacitance, $C_{i}$ is the inner capacitance, k is a constant, and v represents the scan rate.

**S1.3 Deionization measurements**

The deionization experiment was conducted using the batch mode method in a EDI device with activated carbon and prepared materials as the cathode and anode, respectively. The EDI device consisted of a pair of acrylic plates, electrodes, rubber gaskets, and cation/anion exchange membranes. The feed water at a 20 mL min^−1^ flow rate was 40 mL of NaCl solution with a concentration of 1000 mg L^−1^. And the real-time solution conductivity during charged/discharged steps was monitored and recorded by a conductivity meter (METTLER TOLEDO S230, Switzerland). All deionization experiments were performed in constant voltage mode with voltage of 1.0 V, 1.2 V, 1.4 V, and 1.6 V for 30 min. The voltage in the long-term adsorption-desorption cycle was set to 1.2 V for 10 min, and all corresponding desorption processes were performed by applying the same duration of reverse voltage.

The areal deionization capacity (ADC) (mg-Cl^−^ cm^−2^) was calculated according to the equation (S5):

$$\begin{aligned} ADC=\frac{\left( C_{o}-C_{e} \right)\times V\times M}{S}\#\left( S5 \right) \end{aligned}$$

Where *C_o_* and *C_e_* (mg L^−1^) are the concentration of NaCl at initial and final stages, respectively; *V* is the volume of NaCl solution; M represents the molecular weight of Cl^−^ and *S* (cm^2^) is the total mass areal of active material in working electrode.

The time average areal deionization rate (ADR, mg-Cl^−^ cm^−2^ min^−1^) was acquired from the equation (S6).

$$\begin{aligned} ADR=\frac{ADC}{t}\#\left( S6 \right) \end{aligned}$$

Where *ADC* (mg-Cl^−^ cm^−2^) is the areal deionization capacity, *t* is the deionization time (min).

The energy consumption (EC, kWh kg^−^-Cl^−^) was quantitatively determined according to equation (S7)

$$\begin{aligned} \text{EC}\text{= }\frac{\text{U}\int Idt}{\text{ADC}\text{×}\text{S}}\#\left( S7 \right) \end{aligned}$$

Where *U* (V) and $\text{I}$ (A) are applied voltage and current, respectively.

**S1.4 Density functional theory (DFT) calculations**

All the calculations are performed in the framework of the density functional theory with the projector augmented plane-wave method, as implemented in the Vienna ab initio simulation package [S1]. The generalized gradient approximation proposed by Perdew, Burke, and Ernzerhof is selected for the exchange-correlation potential [S2]. The cut-off energy for plane wave is set to 450 eV. The energy criterion is set to 10^−5^ eV in iterative solution of the Kohn-Sham equation. A vacuum layer of 15 Å is added perpendicular to the sheet to avoid artificial interaction between periodic images. The Brillouin zone integration is performed using a 2×2×1 k-mesh. The Van der Waals dispersion-corrected DFT was also carried out, as proposed by Grimme et al [S3]. All the structures are relaxed until the residual forces on the atoms have declined to less than 0.02 eV/Å. The Gibbs free energy change (ΔG) of each elementary step was calculated by the following equation (S8)

$$\begin{aligned} \Delta G=\Delta E+\Delta E_{ZPE}-T\Delta S\#\left( S8 \right) \end{aligned}$$

where ΔE is the electronic energy change between the two intermediates. ΔE_ZPE_ and ΔS refer to the zero-point energy correction and entropy change, respectively. The temperature T corresponds to 298.15 K.

The P vacancy formation energy as calculated by using the equation (S9).

$$\begin{aligned} E_{f}^{vac}=E\left( F-\frac{Cu_{3}P_{v}}{Cu_{3}P_{v}} \right)+E\left( P \right)-E\left( F-\frac{{Cu}_{3}P}{{Cu}_{3}P} \right)\#\left( S9 \right) \end{aligned}$$

**Supplementary Figures and Tables**


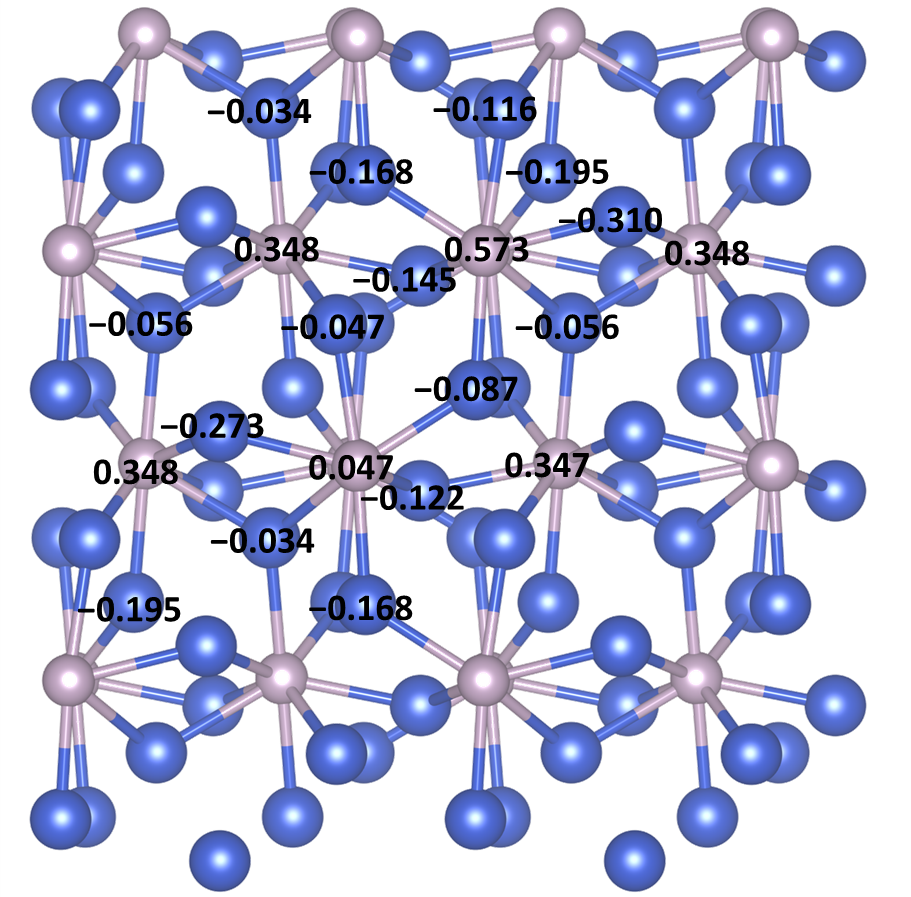


**Fig. S1** The Bader charge numbers of atoms in Cu_3_P.


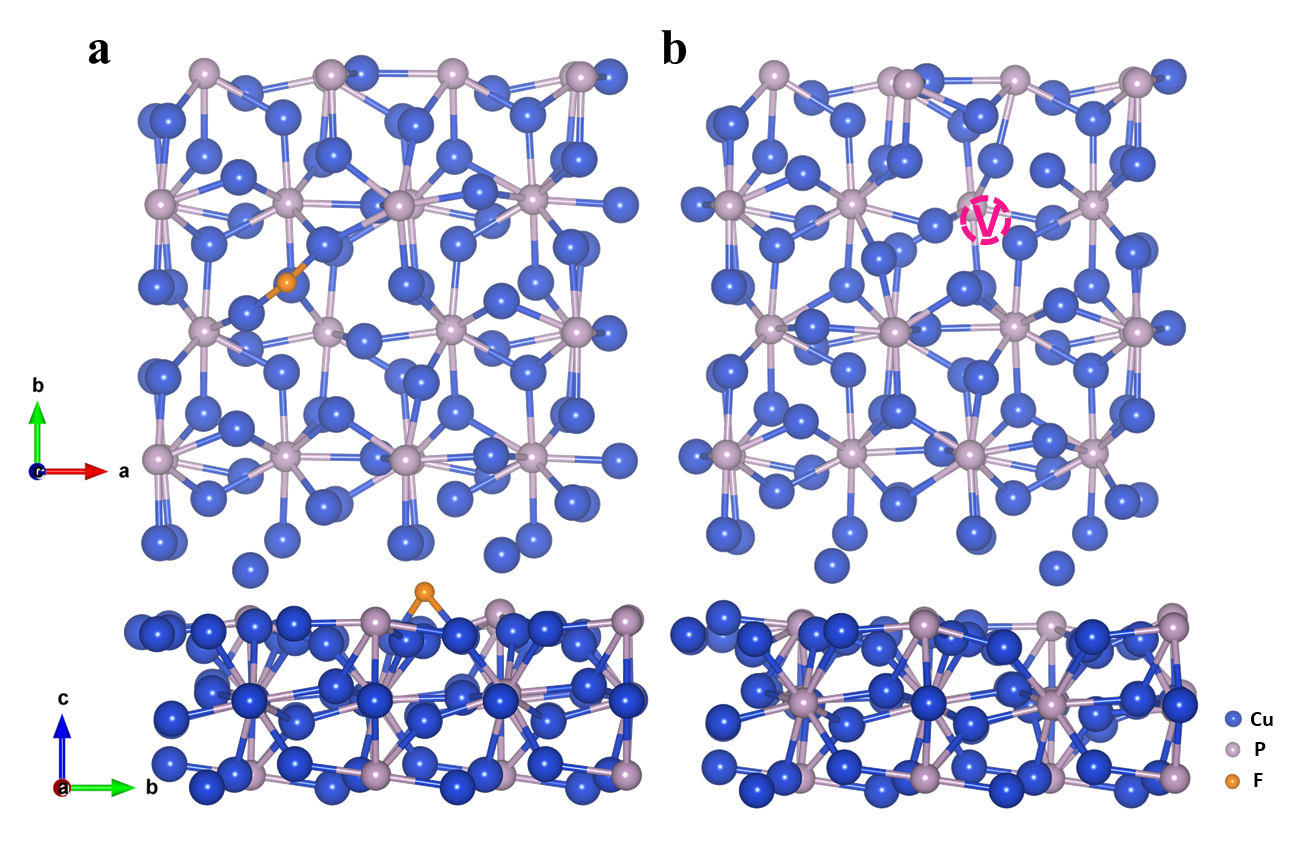


**Fig. S2** The optimized structure of **a** F-Cu_3_P and **b** Cu_3_P_V_ models.


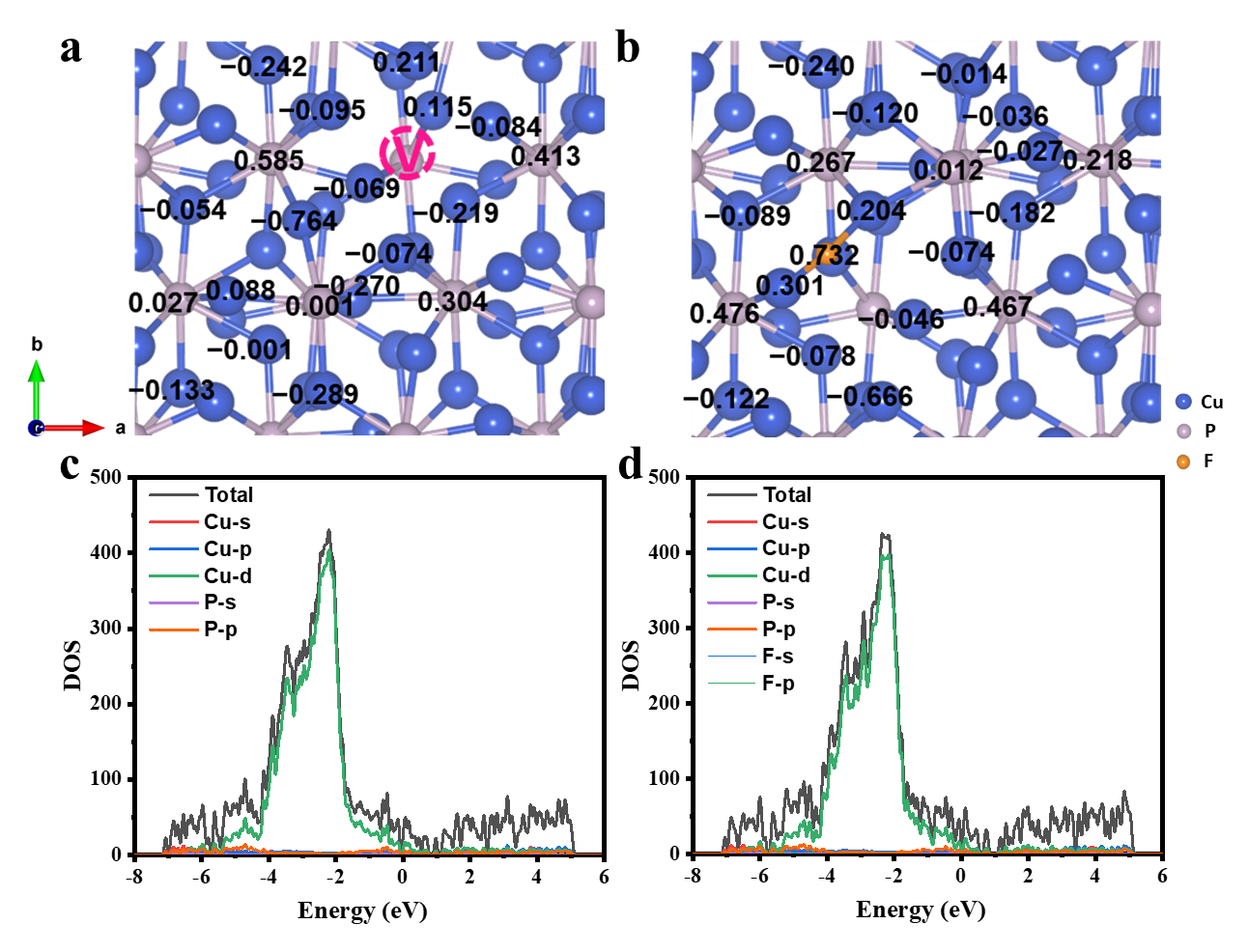


**Fig. S3** The Bader charge numbers of atoms in **a** F-Cu_3_P and **b** Cu_3_P_V_ (Positive and negative values represent the accumulation and depletion of electrons, respectively.). Calculated DOS of **c** F-Cu_3_P and **d** Cu_3_P_V_.

We constructed models with only F doping (F-Cu_3_P) and only P vacancy (Cu_3_P_V_), with their optimized configurations shown in Fig. S2, and compared them with Cu_3_P and F-Cu_3_P_V_ models. The Bader charge results indicate that both F doping and P vacancy induce localized electronic redistribution (Fig. S3a-b). Specifically, F doping, due to high electronegativity, attracts electrons from neighboring Cu atoms, thereby affecting the electron distribution around Cu and P atoms. P vacancy causes electrons originally occupying the P 2p orbitals to delocalize to the Cu atoms around the vacancy, while the electron density around neighboring P atoms is also affected to a certain extent at the same time. In addition, the ordering of the electronic density of states near the Fermi energy level for the four models is as follows: F-Cu_3_P_V_ > Cu_3_P_V_ > F-Cu_3_P > Cu_3_P, indicating that both F doping and P vacancy can improve electrical conductivity, thereby enhancing electron-transfer kinetics (Fig. S3c-d). Moreover, compared with F doping, P vacancy displays a more significant enhancement in electronic conductivity.


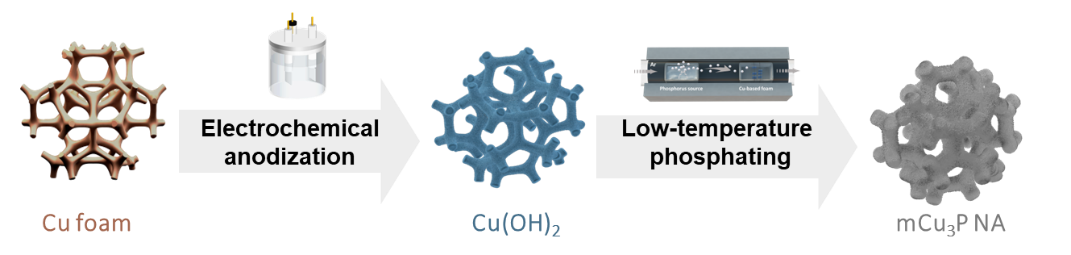


**Fig. S4** Schematic illustration of mCu_3_P NA synthesis process.


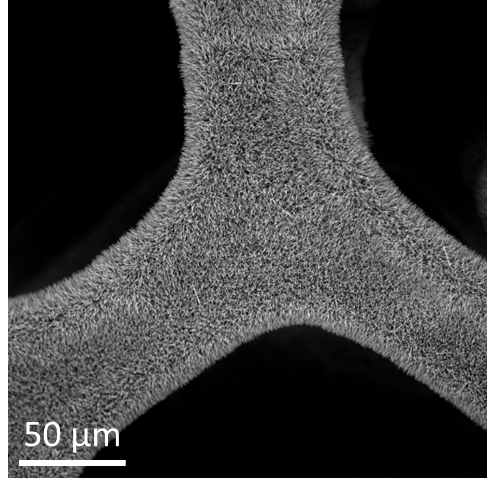


**Fig. S5** SEM image of Cu(OH)_2_ nanowire arrays.


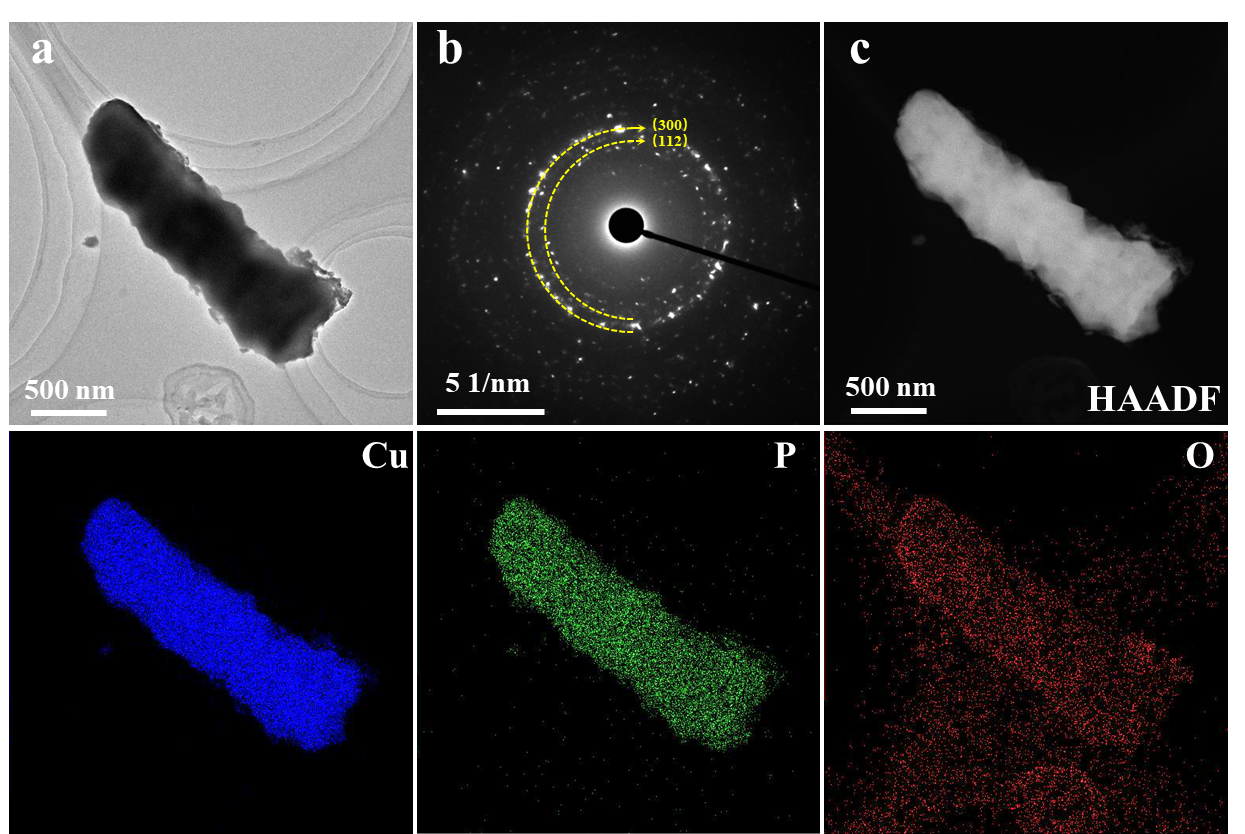


**Fig. S6** **a** TEM image, **b** SEAD, and **c** HAADF scanning TEM image as well as corresponding EDS element mappings of mCu_3_P NA.

**Fig. S7** EPR spectra of prepared materials.

**Fig. S8** The CV curves of F-Cu_3_P_V_-1 and F-Cu_3_P_V_-3 at scan rate of 10 mV s^−1^.


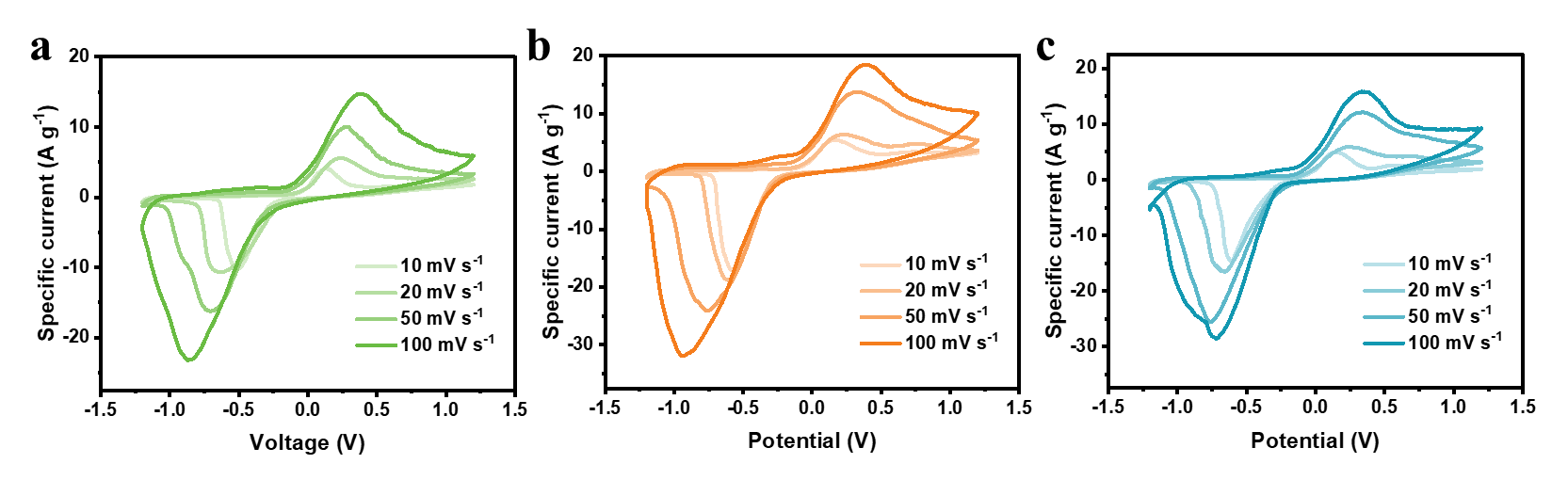


**Fig. S9** The CV curves of **a** mCu_3_P NA, **b** F-Cu_3_P_V_-1, and **c** F-Cu_3_P_V_-3 at different scan rates.

**Fig. S10** Specific capacitances of F-Cu_3_P_V_-1 and F-Cu_3_P_V_-3 at different scan rates.


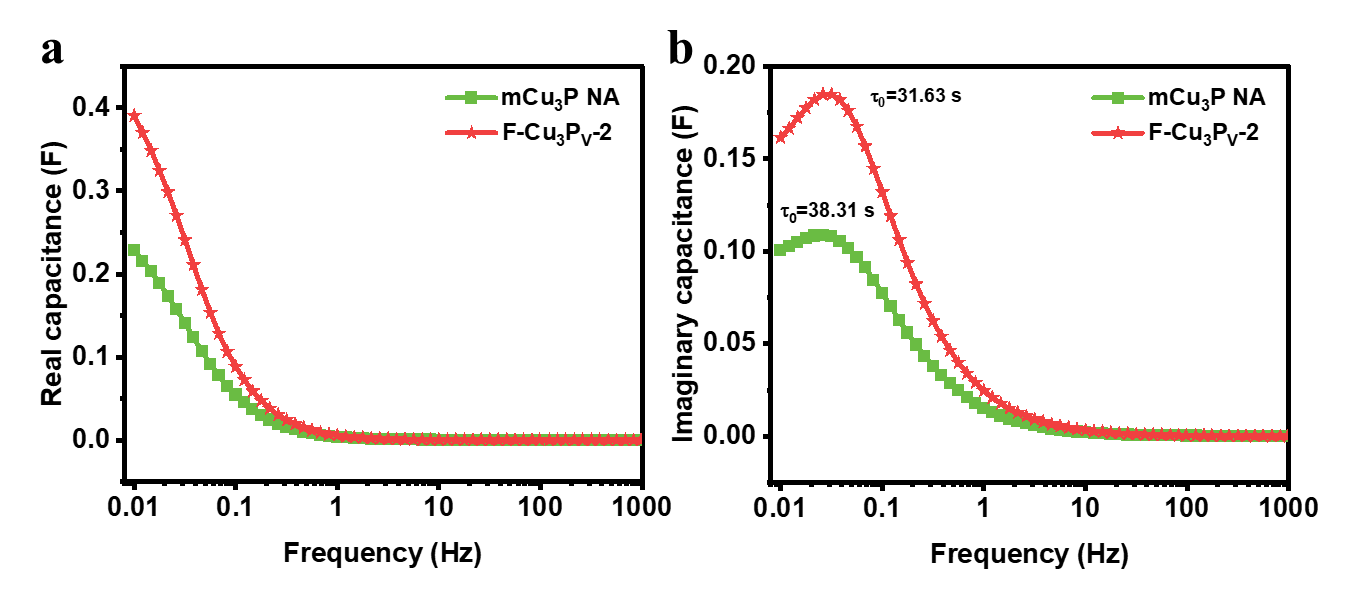


**Fig. S11** **a** The real capacitance and **b** imaginary capacitance against frequency of mCu_3_P NA and F-Cu_3_P_V_-2.


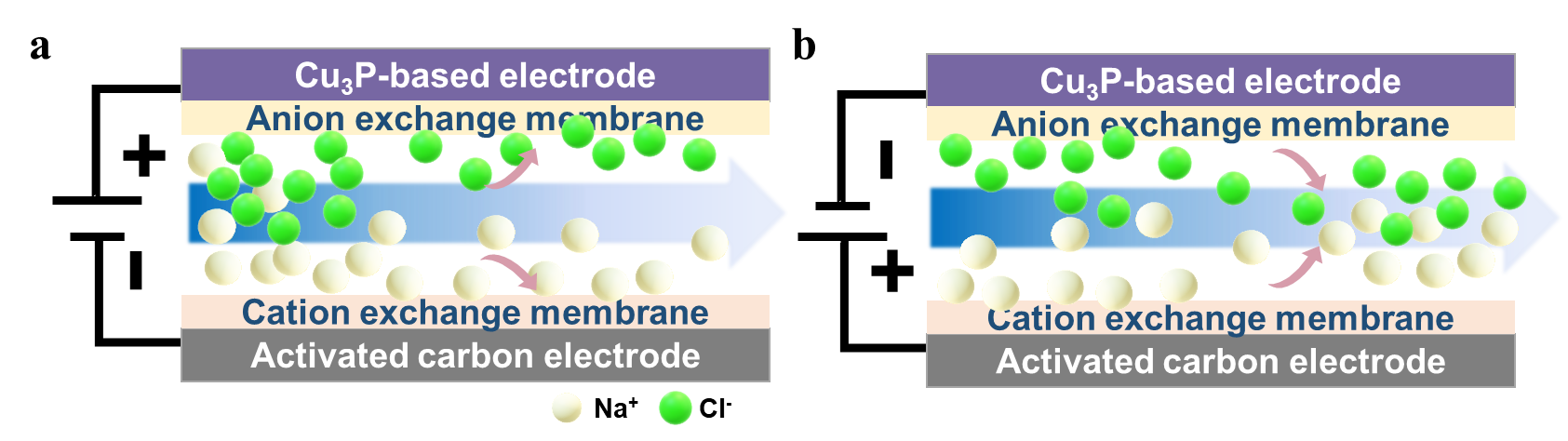


**Fig. S12** Schematic illustrations of **a** capture and **b** release processes in EDI system.

**Fig. S13** Temporal variation in the ADC of prepared materials at 1.2 V.

**Fig. S14** Temporal variation in the ADR of mCu_3_P NA and F-Cu_3_P_V_-2 at 1.2 V.

**Fig. S15** Comparison of energy consumption of electrochemical chloride ion removal.


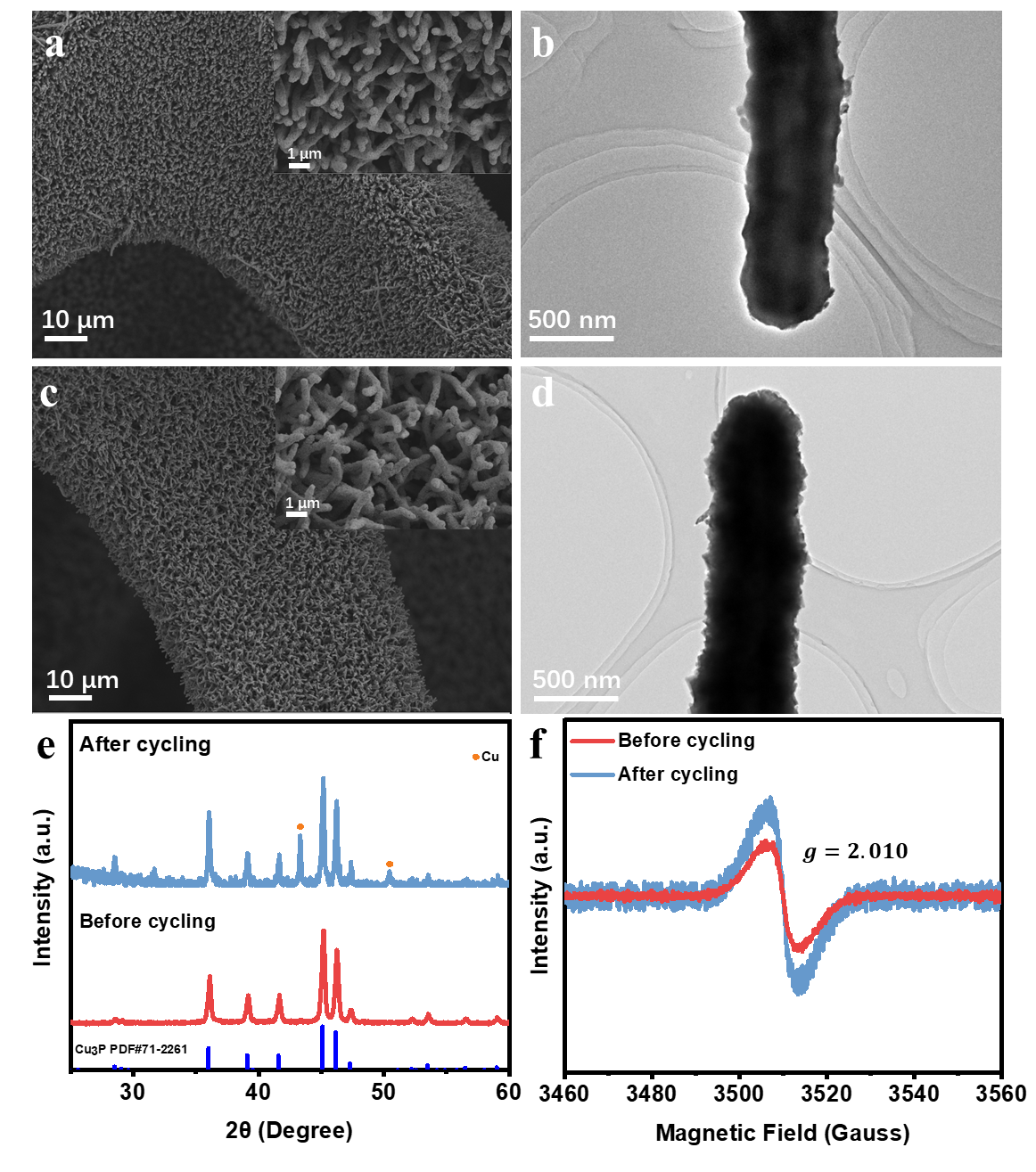


**Fig. S16** SEM images and TEM images of F-Cu_3_P_V_-2 **a-b** before and **c-d** after cycling, **e** XRD, and **f** EPR of F-Cu_3_P_V_-2 before and after cycling.
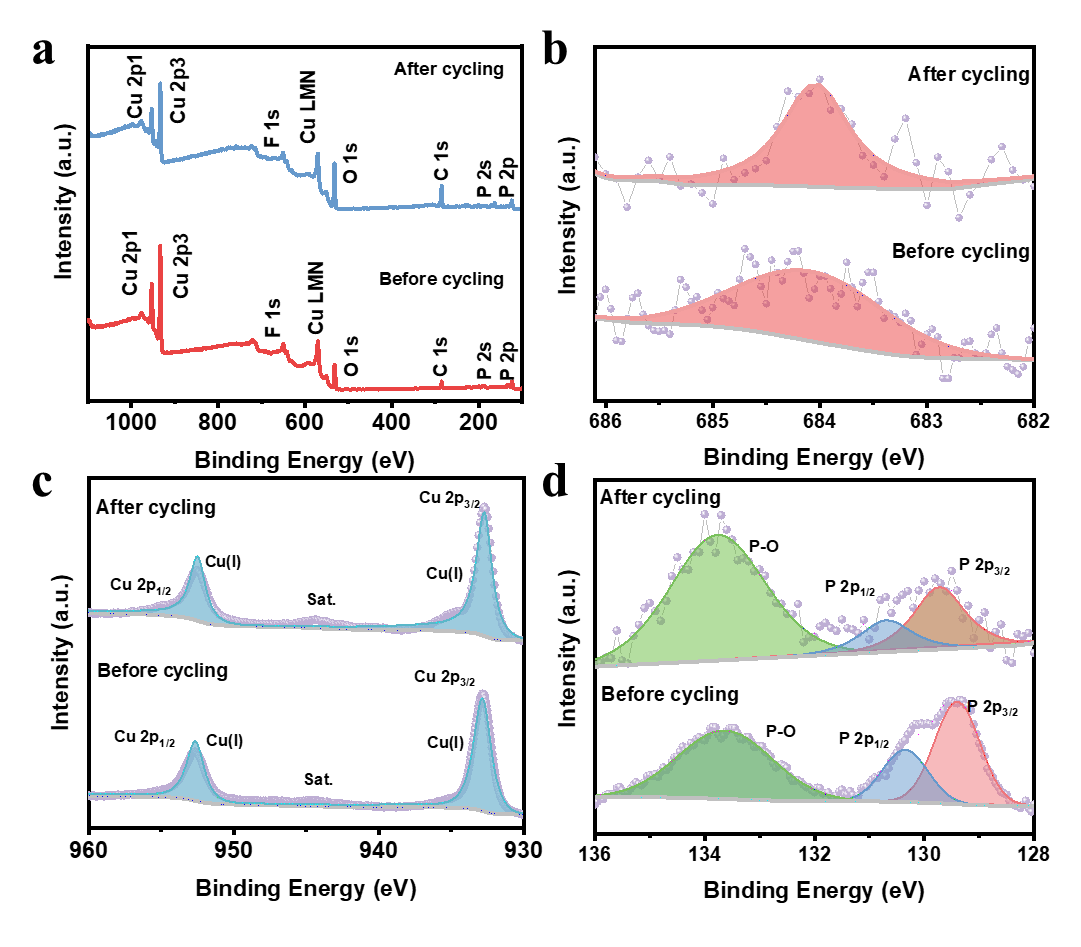


**Fig. S17 a** XPS survey, **b** High-resolution XPS of F 1s, **c** Cu 2p, and **d** P 2p of F-Cu_3_P_V_-2 before and after long-term cycling.


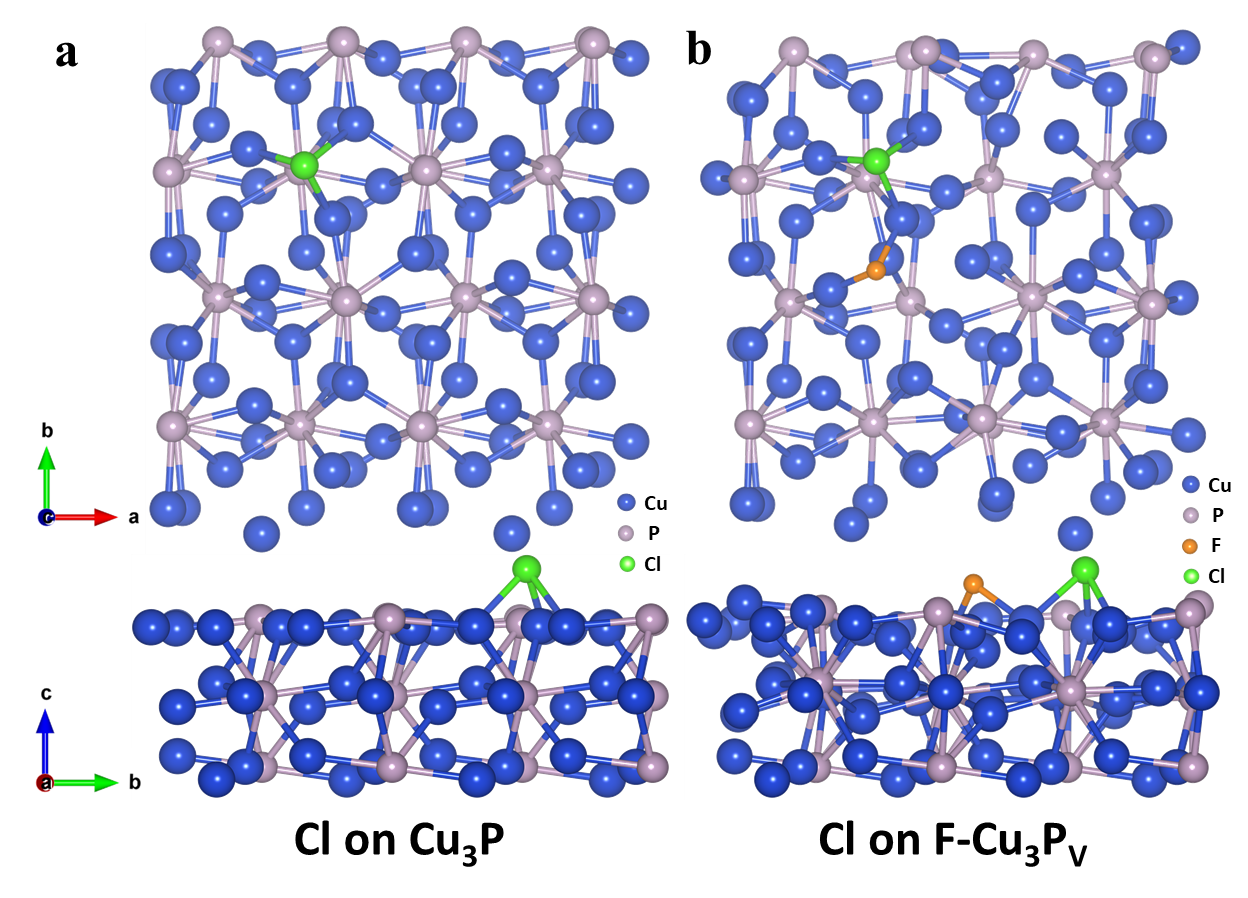


**Fig.** **S18** Optimal structure of Cl on **a** Cu_3_P model and **b** F-Cu_3_P_V_ models.


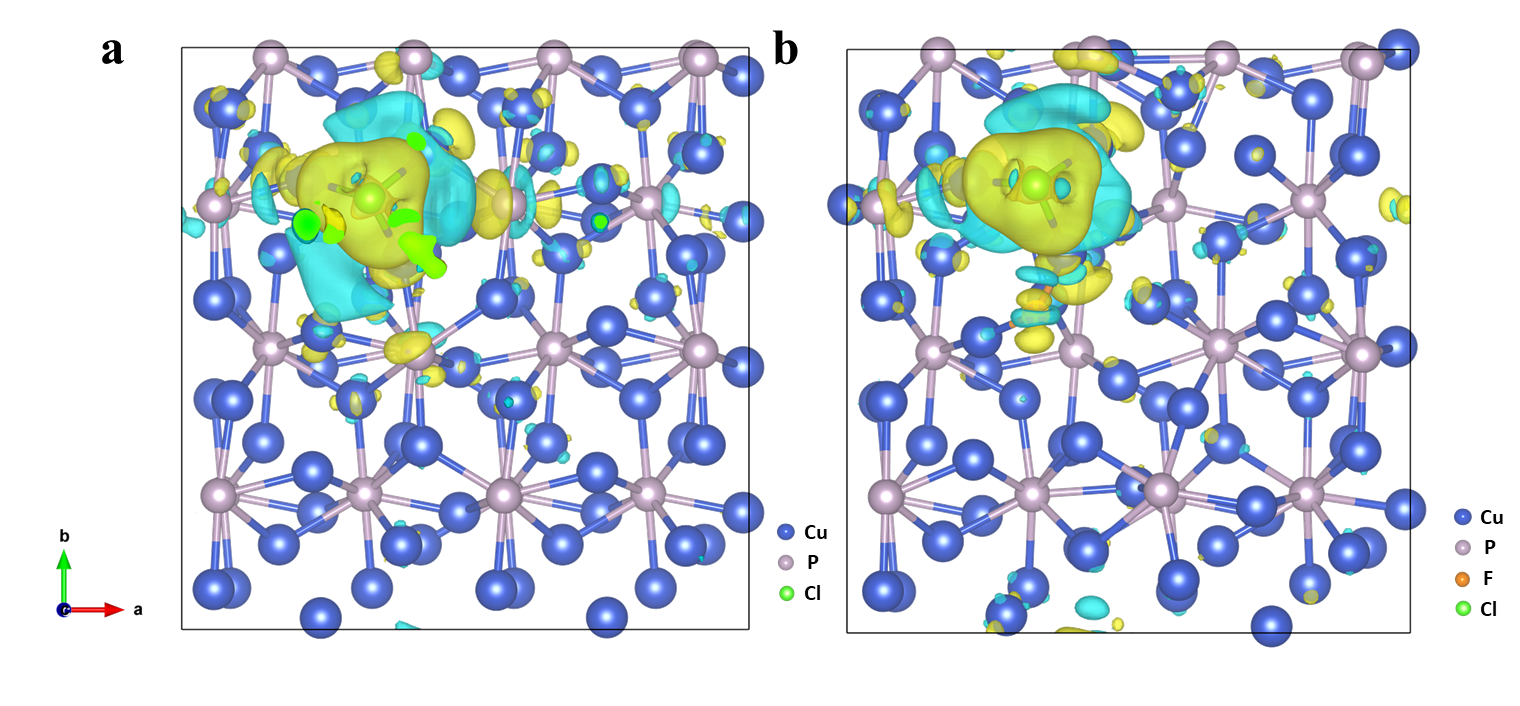


**Fig. S19** Top views of the charge density difference during the Cl^−^ adsorption for **a** Cu_3_P and **b** F-Cu_3_P_V_. (Yellow area indicates electron accumulation and green area indicates electron depletion.)


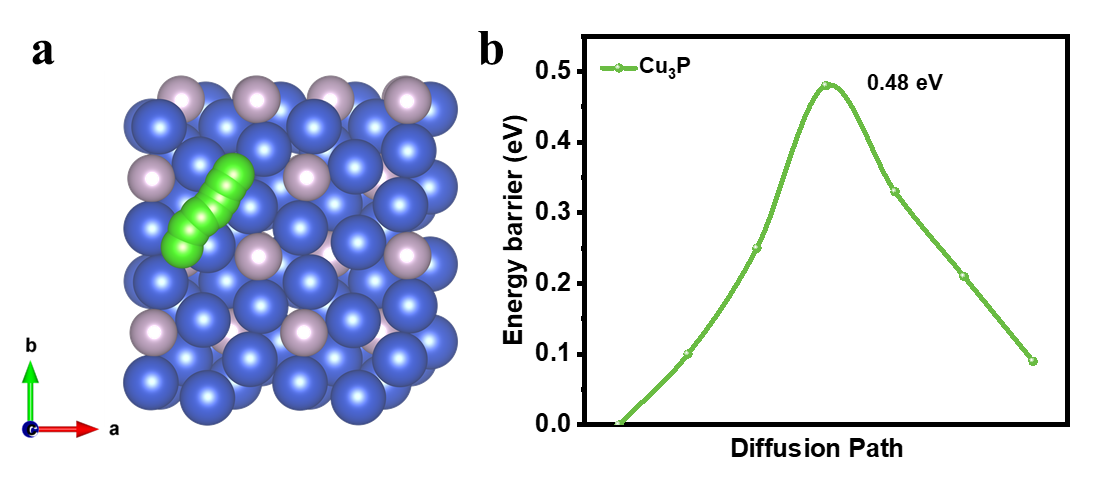


**Fig. S20** **a** Top view of Cl^−^ diffusion path on Cu_3_P and **b** corresponding energy barrier diffusion curves.


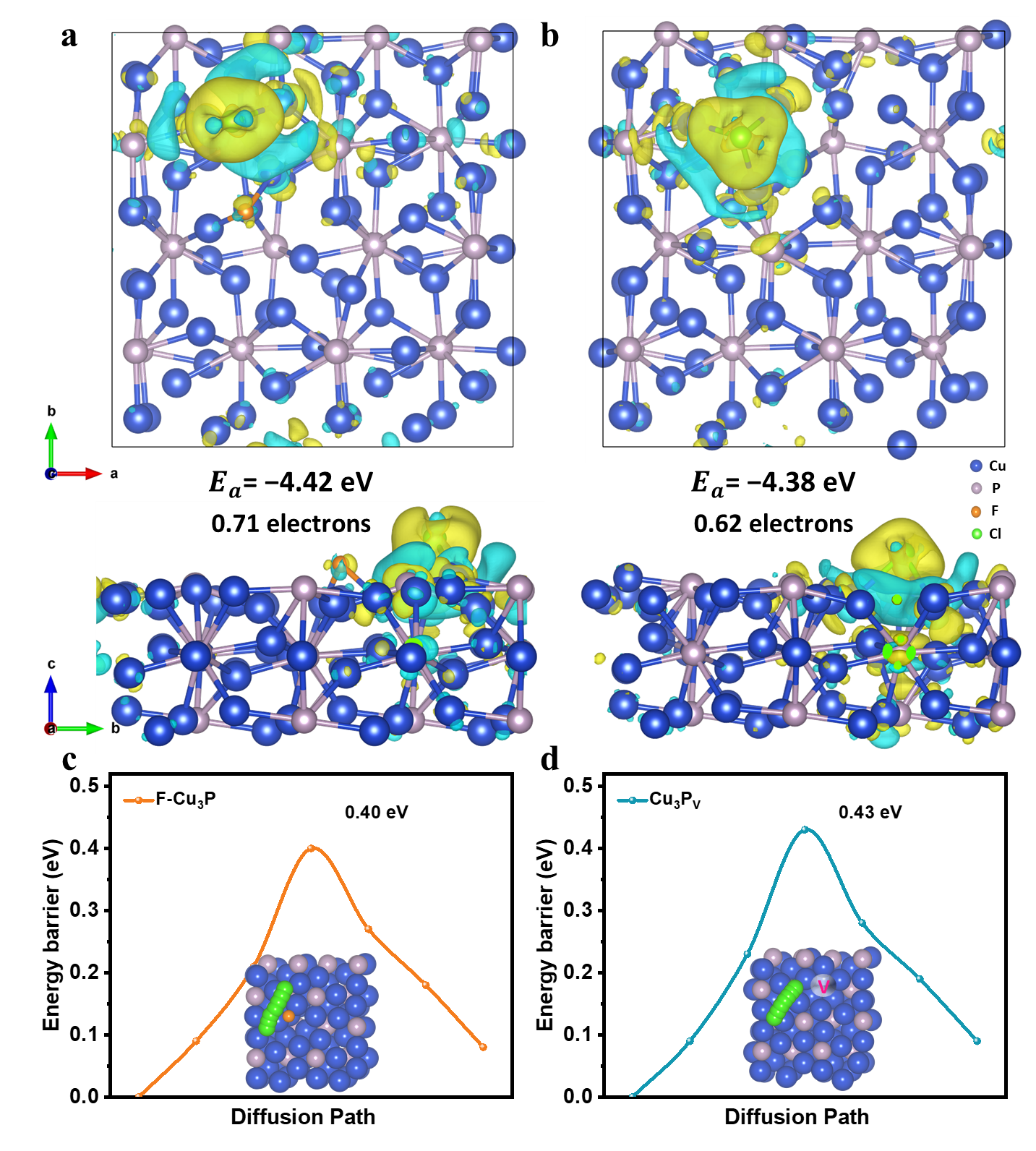


**Fig. S21** Top and front views of the charge density difference during the Cl^−^ adsorption for **a** F-Cu_3_P and **b** Cu_3_P_V_. (Yellow area indicates electron accumulation and green area indicates electron depletion.) Top view of Cl^−^ diffusion path on **c** F-Cu_3_P and **b** Cu_3_P_V_ and corresponding energy barrier diffusion curves.

The F-Cu_3_P model exhibits more significant charge transfer and a more negative adsorption energy (−4.42 eV) compared to the Cu_3_P_V_ model (−4.38 eV), suggesting that F doping shows a greater enhancement on Cl^−^ adsorption than P vacancy. Moreover, the ordering of diffusion energy barriers for the four models is F-Cu_3_P_V_ < F-Cu_3_P < Cu_3_P_V_ < Cu_3_P, indicating that F doping is more effective at optimizing ion diffusion kinetics than P vacancy.

**Table S1** Comparison of areal EDI performance of Cl^−^ removal electrode materials

| **No.** | **Electrodes** | | | **Mass loading (mg cm^−2^)** | **Initial salt concentration (mg L^−1^)** | **Applied voltage/** | **Desalination capacity**  **(mg-Cl^−^ cm^−2^)** | **Deionization Rate**  **(mg-Cl^−^ cm^−2^ min^−1^)** | **Refs.** |
| --- | --- | --- | --- | --- | --- | --- | --- | --- | --- |
|  |  |  |  |  |  | **Current density** |  |  |  |
| 1 | **Carbon materials** | Three-dimensional flexible graphene frameworks | 3DFGFs | 16.80 | 500 | 1.6 | 0.70 | 0.0117 | [S4] |
| 2 |  | Nitrogen-doped carbon obtained from HCl pretreatment | HCl-NC | 0.66 | 1000 | 1.2 | 0.08 | 0.0014 | [S5] |
| 3 |  | 3D graphene nanoscroll-nanosheet aerogels | GSSA | 1.13 | 1000 | 1.2 | 0.04 | 0.0006 | [S6] |
| 4 |  | Block copolymer-based porous carbon fibers | PCFs | 0.73 | 500 | 1.0 | 0.04 | 0.0036 | [S7] |
| 5 |  | 3D N-doped hierarchical porous carbon | 3DNHPC | 3.20 | 500 | 1.2 | 0.09 | 0.0304 | [S8] |
| 6 |  | Porous Carbon Fibers Cloth-CNTs | PCF-CNTs | 12.50 | 100 | 1.2 | 0.06 | 0.0021 | [S9] |
| 7 |  | Hollow carbon nanospheres | HCNSs | 3.80 | 584 | 1.2 | 0.05 | 0.0035 | [S10] |
| 8 |  | Cobalt benzimidazole frameworks derived carbon composites | ZC | 7.44 | 585 | 1.2 | 0.25 | 0.0418 | [S11] |
| 9 |  | Wood carbon framework | WCF | 50.00 | 100 | 1.2 | 0.36 | 0.0020 | [S12] |
| 10 |  | 3D graphene after Nirvana with 2 times | NvGII | 32.40 | 500 | 2.0 | 0.58 | 0.0467 | [S13] |
| 11 |  | Order-in-disorder nanosheet-like carbon | O/D NSLC | 2.22 | 1000 | 1.6 | 0.07 | 0.0022 | [S14] |
| 12 | **Conductive polymers** | Bicontinuous mesoporous polypyrrole cubosomes | BM-PPy | 2.00 | 292.2 | 100 mA g^−1^ | 0.76 | 0.0253 | [S15] |
| 12 |  | Metal-organic framework/polypyrrole hybrid | MOF/PPy | 10.00 | 584 | 1.2 | 0.14 | 0.0138 | [S16] |
| 13 |  | Polyaniline in situ grown on the Porous activated carbon surface composites | PAC/PANI | 5.00 | 500 | 1.2 | 0.17 | 0.0057 | [S17] |
| 14 |  | Amino group-rich conjugated microporous polytriphenylamine | m-PTPA | 8.70 | 500 | 1.4 | 0.50 | 0.0335 | [S18] |
|  |  |  |  | 11.70 | 500 | 1.4 | 0.43 | 0.0284 |  |
| 15 | **Bi/Ag based materials** | Highly dispersed Ag nanocrystals on N-doped holey carbon (Ag@NHC) | Ag@NHC | 6.40 | 1500 | 1.4 | 0.93 | 0.0311 | [S19] |
| 16 |  | Bismuth nanoclusters (Bi NCs) within carbon nano-bundles | Bi NCs@CNBs | 3.13 | 1170 | 600 mA g^−1^ | 0.16 | 0.0319 | [S20] |
| 17 |  | Bi NCs-impregnated 3D carbon nanofiber network | Bi NCs@CNF | 3.34 | 3000 | 220.0 | 0.21 | 0.0175 | [S21] |
| 18 |  | Ag-based MOFs | Ag-MOF | 1.80 | 500 | 1.2 | 0.21 | 0.0052 | [S22] |
| 19 |  | Ag-coated activated carbon | Ag/AC | 15.90 | 585 | 1.2 | 0.45 | 0.0450 | [S23] |
| 20 | **Other materials** | FeOCl/Ti_3_C_2_T_x_ | FeOCl/Ti_3_C_2_T_x_ | 0.94 | 1000 | 1.6 | 0.21 | 0.0035 | [S24] |
| 21 |  | FeOOH hybrid heterostructures with Cl dopants | Cl-FeOOH | 2.27 | 584 | 1.2 | 0.05 | 0.0004 | [S25] |
| 22 |  | Cu_2_O | Cu_2_O | 5.43 | seawater | 200 mA g^−1^ | 1.19 | 0.0149 | [S26] |
| 23 |  | CoNiPS@CF | CoNiPS@CF | 3.00 | 1000 | 1.6 | 0.37 | 0.0061 | [S27] |
| 24 |  | Copper(I) phosphide | Cu_3_P | 32.70 | 1000 | 1.6 | 2.21 | 0.0736 | [S28] |
| 25 |  | F-Cu_3_P_V_-2 | F-Cu_3_P_V_-2 | 33.40 | 1000 | 1.2 | 3.17 | 0.1055 | **This work** |

**Table S2** Comparison of areal EDI performance of Cl^−^ removal high-mass-loading electrode materials

| **No.** | **Electrodes** | **Thickness (mm)** | **Mass loading (mg cm^−2^)** | **ADC (mg-Cl^−^ cm^−2^)** | **GDC (mg-Cl^−^ g^−1^)** | **VDC (mg-Cl^−^ cm^−3^)** | **Refs.** |
| --- | --- | --- | --- | --- | --- | --- | --- |
| 1 | PCF-CNTs | 0.33 | 12.5 | 0.06 | 4.95 | 1.88 | [S9] |
| 2 | WCF | 1.2 | 50 | 0.36 | 6.92 | 3.03 | [S12] |
| 3 | NvGII | 0.6 | 32.4 | 0.58 | 18.03 | 9.73 | [S13] |
| 4 | MOF/Ppy | 1 | 10 | 0.14 | 13.76 | 1.38 | [S16] |
| 5 | m-PTPA | 0.6 | 11.7 | 0.43 | 36.41 | 7.10 | [S18] |
| 6 | Ag/AC | 0.3 | 15.9 | 0.45 | 28.28 | 14.99 | [S23] |
| 7 | Cu_3_P-2 | 0.8 | 32.7 | 2.21 | 67.48 | 27.58 | [S28] |
| 8 | F-Cu_3_P_V_-2 | 0.8 | 33.4 | 3.17 | 94.79 | 39.58 | **This work** |

**Supplementary References**

1. G. Kresse, D. Joubert, From ultrasoft pseudopotentials to the projector augmented-wave method. Phys. Rev. B **59**(3), 1758–1775 (1999). <https://doi.org/10.1103/physrevb.59.1758>
2. J.P. Perdew, K. Burke, M. Ernzerhof, Generalized gradient approximation made simple. Phys. Rev. Lett. **77**(18), 3865–3868 (1996). <https://doi.org/10.1103/physrevlett.77.3865>
3. S. Grimme, J. Antony, S. Ehrlich, H. Krieg, A consistent and accurate *ab initio* parametrization of density functional dispersion correction (DFT-D) for the 94 elements H-Pu. J. Chem. Phys. **132**(15), 154104 (2010). <https://doi.org/10.1063/1.3382344>
4. Q. Liu, X. Li, G. Tan, D. Xiao, Chemical bonding of flexible graphene to carbon paper: a new synthetic paradigm for freestanding electrode with high capacitive deionization performance. Desalination **538**, 115890 (2022). <https://doi.org/10.1016/j.desal.2022.115890>
5. M. Liang, N. Liu, X. Zhang, Y. Xiao, J. Yang et al., A reverse-defect-engineering strategy toward high edge-nitrogen-doped nanotube-like carbon for high-capacity and stable sodium ion capture. Adv. Funct. Mater. **32**(49), 2209741 (2022). <https://doi.org/10.1002/adfm.202209741>
6. W. Kong, X. Ge, X. Lu, Q. Zhang, M. Zhang et al., The simpler the better: ultrafast air-plasma synthesis of 3D crosslinked graphene nanoscroll-nanosheet aerogels at room temperature for capacitive deionization. Small **20**(38), 2402057 (2024). <https://doi.org/10.1002/smll.202402057>
7. T. Liu, J. Serrano, J. Elliott, X. Yang, W. Cathcart et al., Exceptional capacitive deionization rate and capacity by block copolymer-based porous carbon fibers. Sci. Adv. **6**(16), eaaz0906 (2020). <https://doi.org/10.1126/sciadv.aaz0906>
8. M. Shi, X. Hong, C. Liu, H. Qiang, F. Wang et al., Green double organic salt activation strategy for one-step synthesis of N-doped 3D hierarchical porous carbon for capacitive deionization. Chem. Eng. J. **453**, 139764 (2023). <https://doi.org/10.1016/j.cej.2022.139764>
9. C. Zhang, D. Wang, Z. Wang, G. Zhang, Z. Liu et al., Boosting capacitive deionization performance of commercial carbon fibers cloth *via* structural regulation based on catalytic-etching effect. Energy Environ. Mater. **6**(1), e12276 (2023). <https://doi.org/10.1002/eem2.12276>
10. Y. Tang, J. Ding, W. Zhou, S. Cao, F. Yang et al., Design of uniform hollow carbon nanoarchitectures: different capacitive deionization between the hollow shell thickness and cavity size. Adv. Sci. **10**(9), 2206960 (2023). <https://doi.org/10.1002/advs.202206960>
11. S. Cao, T. Chen, S. Zheng, Y. Bai, H. Pang, High-performance capacitive deionization and killing microorganism in surface-water by ZIF-9 derived carbon composites. Small Meth. **5**(12), 2101070 (2021). <https://doi.org/10.1002/smtd.202101070>
12. M. Liu, M. Xu, Y. Xue, W. Ni, S. Huo et al., Efficient capacitive deionization using natural basswood-derived, freestanding, hierarchically porous carbon electrodes. ACS Appl. Mater. Interfaces **10**(37), 31260–31270 (2018). <https://doi.org/10.1021/acsami.8b08232>
13. Y. Li, N. Chen, Z. Li, H. Shao, X. Sun et al., Reborn three-dimensional graphene with ultrahigh volumetric desalination capacity. Adv. Mater. **33**(48), 2105853 (2021). <https://doi.org/10.1002/adma.202105853>
14. M. Liang, Y. Ren, J. Cui, X. Zhang, S. Xing et al., Order-in-disordered ultrathin carbon nanostructure with nitrogen-rich defects bridged by pseudographitic domains for high-performance ion capture. Nat. Commun. **15**, 6437 (2024). <https://doi.org/10.1038/s41467-024-50899-5>
15. L. Xiang, X. Xu, Y. Liu, H. Zhang, R. Xu et al., Curvature-induced ion docking effect in capacitive deionization. Nat. Water **2**(12), 1195–1206 (2024). <https://doi.org/10.1038/s44221-024-00340-4>
16. Z. Wang, X. Xu, J. Kim, V. Malgras, R. Mo et al., Nanoarchitectured metal–organic framework/polypyrrole hybrids for brackish water desalination using capacitive deionization. Mater. Horiz. **6**(7), 1433–1437 (2019). <https://doi.org/10.1039/c9mh00306a>
17. B. Li, Q. Cao, Y. Liu, Y. Sun, X. Ma et al., Polyaniline-decorated porous carbons with engineered meso/macrochannels for high performance capacitive deionization. J. Mater. Chem. A **10**(46), 24905–24914 (2022). <https://doi.org/10.1039/d2ta07929a>
18. W. Kong, X. Lu, X. Ge, Q. Zhang, X. Jin et al., Conjugated microporous polytriphenylamine as a high-performance anion-capture electrode for hybrid capacitive deionization with ultrahigh areal adsorption capacity. J. Mater. Chem. A **12**(32), 21124–21133 (2024). <https://doi.org/10.1039/d4ta04010d>
19. W. Kong, X. Lu, Y. Wang, K. Tan, C. Liu et al., Highly dispersed Ag nanocrystals anchored on N-doped holey carbon aerogel as high-mass-loading electrode for Cl− capture in hybrid capacitive deionization. Small **21**(5), 2409342 (2025). <https://doi.org/10.1002/smll.202409342>
20. Y. Liu, L. Wang, Q. Yao, X. Gao, X. Du et al., *In situ* synthesis of bismuth nanoclusters within carbon nano-bundles from metal–organic framework for chloride-driven electrochemical deionization. Adv. Funct. Mater. **32**(12), 2110087 (2022). <https://doi.org/10.1002/adfm.202110087>
21. L. Wang, Z. Liu, Z. Wang, Q. Ma, Z. Guo et al., Up-shifting the desalination rate limit of capacitive deionization *via* integrating chloride-capturing Bi nanocluster with flow-through cell architecture. Chem. Eng. J. **460**, 141726 (2023). <https://doi.org/10.1016/j.cej.2023.141726>
22. D. Wei, B. Ouyang, Y. Cao, L. Yan, B. Wu et al., Coordination confined silver-organic framework for high performance electrochemical deionization. Adv. Sci. **11**(28), 2401174 (2024). <https://doi.org/10.1002/advs.202401174>
23. H. Yoon, J. Lee, T. Min, G. Lee, M. Oh, High performance hybrid capacitive deionization with a Ag-coated activated carbon electrode. Environ. Sci.: Water Res. Technol. **7**(7), 1315–1321 (2021). <https://doi.org/10.1039/d1ew00209k>
24. J. Lei, X. Zhang, J. Wang, F. Yu, M. Liang et al., Interlayer structure manipulation of FeOCl/MXene with soft/hard interface design for safe water production using dechlorination battery deionization. Angew. Chem. Int. Ed. **63**(28), e202401972 (2024). <https://doi.org/10.1002/anie.202401972>
25. J. Zhao, B. Wu, X. Huang, Y. Sun, Z. Zhao et al., Efficient and durable sodium, chloride-doped iron oxide-hydroxide nanohybrid-promoted capacitive deionization of saline water *via* synergetic pseudocapacitive process. Adv. Sci. **9**(25), 2201678 (2022). <https://doi.org/10.1002/advs.202201678>
26. S. Yang, X. Gu, X. Feng, R. Wang, X.-Y. Lou et al., Engineering the reversible redox electrochemistry on cuprous oxide for efficient chloride ion uptake. Nat. Commun. **16**, 2282 (2025). <https://doi.org/10.1038/s41467-025-57605-z>
27. S. Xing, N. Liu, Q. Li, M. Liang, X. Liu et al., Reactive P and S Co-doped porous hollow nanotube arrays for high performance chloride ion storage. Nat. Commun. **15**, 4951 (2024). <https://doi.org/10.1038/s41467-024-49319-5>
28. Z. Zhou, Y. Ren, F. Yu, J. Ma, Monolithic low-tortuous copper(I) phosphide nanorod arrays for exceptional areal performance in electrochemical chloride ion removal. Nano Energy **137**, 110792 (2025). <https://doi.org/10.1016/j.nanoen.2025.110792>
